# Global Prevalence of Diabetic Retinopathy in Pediatric Type 2 Diabetes: A Systematic Review and Meta-analysis

**DOI:** 10.1001/jamanetworkopen.2023.1887

**Published:** 2023-03-17

**Authors:** Milena Cioana, Jiawen Deng, Ajantha Nadarajah, Maggie Hou, Yuan Qiu, Sondra Song Jie Chen, Angelica Rivas, Parm Pal Toor, Laura Banfield, Lehana Thabane, Varun Chaudhary, M. Constantine Samaan

**Affiliations:** 1 Department of Pediatrics, McMaster University, Hamilton, Ontario, Canada; 2 Division of Pediatric Endocrinology, McMaster Children's Hospital, Hamilton, Ontario, Canada; 3 Michael G. De Groote School of Medicine, McMaster University, Hamilton, Ontario, Canada; 4 Health Sciences Library, McMaster University, Hamilton, Ontario, Canada; 5 Department of Health Research Methods, Evidence and Impact, McMaster University, Hamilton, Ontario, Canada; 6 Department of Anesthesia, McMaster University, Hamilton, Ontario, Canada; 7 Centre for Evaluation of Medicines, St Joseph’s Health Care, Hamilton, Ontario, Canada; 8 Biostatistics Unit, St Joseph's Healthcare, Hamilton, Ontario, Canada; 9 Division of Ophthalmology, Department of Surgery, McMaster University, Hamilton, Ontario, Canada

## Abstract

**Importance:**

Type 2 diabetes (T2D) is increasing globally. Diabetic retinopathy (DR) is a leading cause of blindness in adults with T2D; however, the global burden of DR in pediatric T2D is unknown. This knowledge can inform retinopathy screening and treatments to preserve vision in this population.

**Objective:**

To estimate the global prevalence of DR in pediatric T2D.

**Data Sources:**

MEDLINE, Embase, the Cumulative Index to Nursing and Allied Health Literature (CINAHL), the Cochrane Library, the Web of Science, and the gray literature (ie, literature containing information that is not available through traditional publishing and distribution channels) were searched for relevant records from the date of database inception to April 4, 2021, with updated searches conducted on May 17, 2022. Searches were limited to human studies. No language restrictions were applied. Search terms included *diabetic retinopathy*; *diabetes mellitus, type 2*; *prevalence studies*; and *child*, *adolescent*, *teenage*, *youth*, and *pediatric*.

**Study Selection:**

Three teams, each with 2 reviewers, independently screened for observational studies with 10 or more participants that reported the prevalence of DR. Among 1989 screened articles, 27 studies met the inclusion criteria for the pooled analysis.

**Data Extraction and Synthesis:**

This systematic review and meta-analysis followed the Meta-analysis of Observational Studies in Epidemiology (MOOSE) and the Preferred Reporting Items for Systematic Reviews and Meta-analyses (PRISMA) reporting guidelines for systematic reviews and meta-analyses. Two independent reviewers performed the risk of bias and level of evidence analyses. The results were pooled using a random-effects model, and heterogeneity was reported using χ^2^ and *I*^2^ statistics.

**Main Outcomes and Measures:**

The main outcome was the estimated pooled global prevalence of DR in pediatric T2D. Other outcomes included DR severity and current DR assessment methods. The association of diabetes duration, sex, race, age, and obesity with DR prevalence was also assessed.

**Results:**

Among the 27 studies included in the pooled analysis (5924 unique patients; age range at T2D diagnosis, 6.5-21.0 years), the global prevalence of DR in pediatric T2D was 6.99% (95% CI, 3.75%-11.00%; *I*^2^ = 95%; 615 patients). Fundoscopy was less sensitive than 7-field stereoscopic fundus photography in detecting retinopathy (0.47% [95% CI, 0%-3.30%; *I*^2^ = 0%] vs 13.55% [95% CI, 5.43%-24.29%; *I*^2^ = 92%]). The prevalence of DR increased over time and was 1.11% (95% CI, 0.04%-3.06%; *I*^2^ = 5%) at less than 2.5 years after T2D diagnosis, 9.04% (95% CI, 2.24%-19.55%; *I*^2^ = 88%) at 2.5 to 5.0 years after T2D diagnosis, and 28.14% (95% CI, 12.84%-46.45%; *I*^2^ = 96%) at more than 5 years after T2D diagnosis. The prevalence of DR increased with age, and no differences were noted based on sex, race, or obesity. Heterogeneity was high among studies.

**Conclusions and Relevance:**

In this study, DR prevalence in pediatric T2D increased significantly at more than 5 years after diagnosis. These findings suggest that retinal microvasculature is an early target of T2D in children and adolescents, and annual screening with fundus photography beginning at diagnosis offers the best assessment method for early detection of DR in pediatric patients.

## Key Points

**Question:**

What is the prevalence of diabetic retinopathy (DR) in children with type 2 diabetes (T2D)?

**Findings:**

In this systematic review and meta-analysis of 27 observational studies including 5924 unique patients with pediatric T2D, 6.99% of participants with T2D had DR; the prevalence increased significantly more than 5 years after T2D diagnosis. The heterogeneity was high across studies.

**Meaning:**

These results suggest that the increasing risk of DR in children with T2D warrants the implementation of global screening programs at diagnosis and annually to ensure early detection and treatment to preserve vision in this population.

## Teaser

This systematic review and meta-analysis uses data from observational studies to estimate the global prevalence of diabetic retinopathy among patients with pediatric type 2 diabetes.

## Introduction

The obesity epidemic has been the primary factor in the increase in pediatric type 2 diabetes (T2D) case numbers globally.^1,2,3,4,5,6,7,8^ Type 2 diabetes is a more aggressive disorder in youths than it is in adults, with early comorbidities and complications including hypertension, nephropathy, polycystic ovary syndrome, and dyslipidemia.^9,10,11,12,13,14,15^

Diabetic retinopathy (DR) is the leading cause of blindness in adults with T2D and has several subtypes.^16,17^ Hyperglycemia increases vascular permeability and can lead to capillary occlusion, which results in nonproliferative DR (NPDR). This phase may be followed by a proliferative phase of DR with the formation of new blood vessels. Macular edema with fluid accumulation can also develop and may impact central vision.^18^

Children are developing T2D early in life and will live with their diabetes for several decades, which may increase their lifetime risk of developing DR and progress to blindness if undetected and untreated.^19,20^ While current guidelines recommend screening for DR in youths with T2D at diagnosis and annually thereafter, the global burden of DR is still not fully quantified.^21,22^ Understanding the scale of DR will help define its natural history and support the development of personalized clinical practice guidelines dedicated to children with T2D.

The main goal of this systematic review and meta-analysis was to assess the global prevalence of DR in pediatric patients with T2D. We also aimed to assess the severity profile of DR and the current diagnostic assessment methods. Other outcomes included the association of diabetes duration, sex, race, age, obesity, hypertension, and hemoglobin A_1c_ (HbA_1c_) level with DR prevalence.

## Methods

### Systematic Review Protocol and Registration

This systematic review and meta-analysis has been registered with the International Prospective Register of Systematic Reviews (PROSPERO; CRD42018091127).^23^ The study was exempt from the need for review and approval by an ethics review board because we used only aggregated deanonymized data that were already published. This study followed the Meta-analysis of Observational Studies in Epidemiology (MOOSE)^24^ and the Preferred Reporting Items for Systematic Reviews and Meta-analyses (PRISMA)^25^ reporting guidelines for systematic reviews and meta-analyses (eTable 1 in Supplement 1).

### Search Strategies

A senior health sciences librarian (L.B.) developed the search strategies in MEDLINE, Embase, the Cumulative Index to Nursing and Allied Health Literature (CINAHL), the Cochrane Central Register of Controlled Trials, the Cochrane Database of Systematic Reviews, and the Web of Science: Conference Proceedings Citation Index–Science. We searched the gray literature (ie, literature containing information produced by government agencies, academic institutions, and for-profit organizations that is not available through traditional publishing and distribution channels) through access to ClinicalTrials.gov, the Cochrane Central Registry of Controlled Trials, the Web of Science: Conference Proceedings Citation Index–Science, and article references screened at the full-text stage to retrieve potentially eligible articles (eTables 2-6 in Supplement 1). Concepts of pediatrics and T2D were combined with terms referencing observational study design and DR. Search terms included *diabetic retinopathy*; *diabetes mellitus, type 2*; *prevalence studies*; and *child*, *adolescent*, *teenage*, *youth*, and *pediatric*. No language restrictions were applied, and the searches were limited to human studies. If a conference abstract was suitable for inclusion, we searched for full-text articles in the included databases. We also contacted the principal investigators to determine whether the studies were published and to obtain data as needed. The initial database searches were from the date of database inception to April 4, 2021, with updated searches conducted on May 17, 2022.

### Eligibility Criteria

The T2D diagnostic criteria used by different studies are reported in eTable 7 in Supplement 1. In our original protocol, eligible studies included patients with T2D who were diagnosed at 18 years or younger, reported findings for 10 or more patients, had an observational study design (including cross-sectional and cohort studies), and reported the prevalence of DR. During screening, we identified a substantial number of studies that defined pediatric populations as 21 years or younger, so this age cutoff was adopted for study inclusion. We excluded articles reporting on patients with gestational diabetes or other types of diabetes. We planned to include the largest reported sample size for large studies with serial data reporting.

### Study Selection, Data Abstraction, and Quality Appraisal

Three teams of 2 independent reviewers (including M.C., J.D., A.N., M.H., Y.Q., S.S.J.C., A.R., and P.P.T.) screened titles, abstracts, and full-text articles, completed data abstraction, and assessed the risk of bias and level of evidence. Disagreements were resolved through discussion, and a third reviewer (M.C.S.) was available for consultation and resolution of ongoing disputes. The extracted data included age at diagnosis and study inclusion, sex, race, diabetes duration, sample size, obesity rates, method of DR diagnosis, DR classification, total prevalence of DR, and sex- and race-specific prevalence proportions (if reported). Race-based data were collected because race-based differences in T2D prevalence and DR rates were previously reported.^26,27^ Race-specific data from the studies were obtained either from medical records or self-reported information from participants. If a longitudinal study reported the prevalence of DR at multiple time points, we extracted the values closest to the time of diabetes diagnosis. We also contacted the principal investigators of the studies to retrieve missing data when needed.

The risk of bias was assessed using a validated instrument developed for prevalence studies that evaluates the internal and external validity of studies.^28^ The level of evidence was evaluated using the Oxford Centre for Evidence-Based Medicine criteria.^29^ Local and current random sample surveys were given a level of 1 and nonrandom surveys a level of 3 (corresponding to the highest and lowest levels of evidence used in this systematic review and meta-analysis); studies were also rated lower based on imprecision, indirectness, and inconsistency.^29^

### Statistical Analysis

We performed a random-effects meta-analysis if 2 or more studies reported on the prevalence of DR in similar populations using identical study designs, methods, and outcomes.^30,31^ Otherwise, we tabulated the results and presented a narrative review of the studies. The primary outcome was the global pooled prevalence (with 95% CI) of DR. We conducted the meta-analysis with prevalence estimates transformed using the Freeman-Tukey double arcsine method^31^ to prevent the need to stabilize variances because some studies reported prevalence rates of 0%, and we transformed the results back to prevalence estimates for interpretation.^32,33^ To verify the results of the Freeman-Tukey double arcsine analysis and to control for sampling error and bias, an exploratory analysis was also conducted using the random intercept mixed-effects logistic regression model, recognizing that the model does not account for study weights.^34^ In addition to 95% CIs, we also estimated 95% prediction intervals (PIs) to assess the possible range of new values in the present study.^35^ Both inconsistency index (*I*^2^ statistic) and *χ^2^ P* values were used to quantify heterogeneity. An *I*^2^ greater than 75% and *P* < .10 were indicators of heterogeneity.^36^

Subgroup analyses, meta-regression analysis, sensitivity analysis, and publication bias evaluations were performed only if more than 10 studies were identified for a given outcome. Subgroup meta-analyses were performed when 2 or more studies reported the prevalence of DR by sex or race, with the latter classified using National Institutes of Health definitions.^37^ We also performed subgroup analyses by DR severity classification (minimal to moderate NPDR, severe NPDR, proliferative DR, and macular edema), assessment method (fundoscopy or fundus photography), and diabetes duration (<2.5 years, 2.5-5.0 years, or >5.0 years). We also added a random-effects meta-regression analysis to assess the separate associations of obesity, hypertension, HbA_1c_ level, age at diagnosis, age at study inclusion, and diabetes duration with DR prevalence.^36^ We reported the statistical significance of the regression coefficient for the association between each variable and DR prevalence. We calculated the mean difference in HbA_1c_ level for patients with vs without DR. Sensitivity analysis was performed by removing studies reported only in conference abstracts, studies with a sample size of 50 patients or less, studies that included some participants older than 18 years, or studies with a high risk of bias. Publication bias assessment was conducted with a contour-enhanced funnel plot and Egger test, and visual inspection was used to assess asymmetry.^38^ The meta-analysis of prevalence was performed using the metafor package in RStudio software, version 1.1.383, using R language version 3.4.3 (R Foundation for Statistical Computing).^39,40,41^

## Results

### Study Selection and Characteristics

We screened 1989 deduplicated titles and abstracts, and 190 abstracts were chosen for full-text screening. A total of 29 studies^12,42,43,44,45,46,47,48,49,50,51,52,53,54,55,56,57,58,59,60,61,62,63,64,65,66,67,68,69^ met the inclusion criteria (eFigure 1 in Supplement 1). Of those, 6 studies^42,43,44,45,46,47^ (20.7%) had a cross-sectional design, 13 studies^48,49,50,51,52,53,54,55,56,57,58,59,60^ (44.8%) had a retrospective cohort design, and 10 studies^12,61,62,63,64,65,66,67,68,69^ (34.5%) had a prospective cohort design. Additional details about the included studies are reported in the Table and eTable 8 in Supplement 1.

**Table.** Studies Included in the Systemic Review and Meta-analysis

Source	Study location	Study design	Age, y		Duration of diabetes, y	Prevalence of DR, No. (%)	Sample size	Diabetic retinopathy classification: No. (%) of participants	Method of assessment	Definition
			Diagnosis of T2D	Study enrollment						
Eppens et al,^42^ 2006	Western Pacific	Cross-sectional	Median (IQR), 12.0 (10.7 to 13.5)^a^	Median (IQR), 14.9 (13.2 to 16.4)^a^	Median (IQR), 2.3 (1.4 to 3.6)^a^	2 (0.6)	284	NR	NR	NR
Farah et al,^43^ 2006	US	Cross-sectional	<21.0	Range, 10.0 to 21.0^a^	Median (range), 1.8 (<2.0 to 15.0)^a^	1 (2.5)	40	Minimal NPDR: 1 (2.5)	Fundoscopy	Modified Airlie House
Unnikrishnan et al,^44^ 2008	India	Cross-sectional	Mean (SD), 16.2 (2.9)	Mean (SD), 18.9 (4.9)	NR	0	36	NR	Fundoscopy	Background or proliferative retinopathy
Aulich et al,^45^ 2019	Australia	Cross-sectional	NR	Mean (SD), 15.1 (1.9)^a^	Median (IQR), 1.8 (0.3 to 3.3)^a^	2 (6.7)	30	NR	7-Field stereoscopic fundus photography	ETDRS
Khalil et al,^46^ 2019	Egypt	Cross-sectional	Mean (SD), 18.0 (2.0)	Mean (SD), 19.8 (1.1)	Mean (SD), 2.5 (2.0)	0	13	NR	Fundoscopy	Normal fundus, nonproliferative retinopathy, or proliferative retinopathy
Ferm et al,^47^ 2021	US	Cross-sectional	NR	NR	NR	13 (3.1)	416	NR	Nonmydriatic fundus photography	NR
Scott et al,^48^ 2006	New Zealand	Retrospective cohort	NR	Mean (SD), 20.0 (0.4)	Mean (SD), 3.0 (0.3)	8 (7.6)	105	Mild to moderate NPDR: 4 (3.8); severe NPDR, PDR, or macular edema: 4 (3.8)	Either fundoscopy or fundus photography (type NR)	NR
Lee et al,^49^ 2007	Japan	Retrospective cohort	NR	NR	NR	10 (27.8)	36	NR	NR	NR
Osman et al,^50^ 2013	Sudan	Retrospective cohort	<10 y: 3 participants; 11 to 18 y: 35 participants	NR	NR	0	38	NR	NR	Medical records
Dart et al,^51^ 2014	Canada	Retrospective cohort	Mean (SD), 13.5 (2.2)	Mean (SD), 16.5 (2.3)	Median (range), 4.4 (0 to 27.4)	40 (11.7)	342	NR	NR	Medical records
Geloneck et al,^52^ 2015	US	Retrospective cohort	Mean (SD), 11.8 (2.7)	Mean (SD), 14.5 (2.1)	Mean (SD), 2.8 (2.3)	0	32	NR	Fundoscopy	Medical records
Newton et al,^53^ 2015^b^	New Zealand	Retrospective cohort	Range, 6.5 to 17.0^c^	<17.0	0	1 (4.3)	23	NR	NR	Medical records
Wang et al,^54^ 2017	US	Retrospective cohort	Median (IQR), 18.0 (16.0 to 21.0)	NR	Median (IQR), 3.1 (1.9 to 4.9)	127 (7.2)	1768	NPDR: 6 (0.3); PDR: 1 (0.1); unspecified: 120 (6.8)	NR	Medical records
Yeh and Bernardo,^55^ 2017^b^	US	Retrospective cohort	Mean, 13.8	NR	NR	1 (7.1)	14	NR	Fundoscopy	Medical records
Koziol et al,^56^ 2020	Poland	Retrospective cohort	NR	<18.0	NR	79 (1.8)	4291	NR	NR	Medical records
Ek et al,^57^ 2020	Sweden	Retrospective cohort	Mean (SD), 15.0 (1.9)^a^	Mean (SD), 22.2 (3.7)	Mean (SD), 6.7 (2.8)	32 (31.1)	103	NR	Fundus photography (type NR)	NR
Porter et al,^58^ 2020	US	Retrospective cohort	Mean (SD), 17.0 (3.0)	Mean (SD), 18.1 (2.6)	Mean (SD), 1.1 (1.3)	3 (6.0)	50	Mild NPDR: 3 (6.0)	NR	ETDRS
Amutha et al,^59^ 2021	India	Retrospective cohort	Mean (SD), 16.6 ( 2.5)^a^	Mean (SD), 23.2 ( 9.7)^a^	Median (IQR), 5.7 (NR to NR)^a^	118 (27.5)	429	NR	Both fundoscopy (for initial screening) and 7-field stereoscopic fundus photography (for confirmation)	ETDRS
Bai et al,^60^ 2022	US	Retrospective cohort	Mean (SD), 17.3 (3.4)	<22.0	Range, 0 to 15.0	17 (26.6)	64	NPDR: 11 (64.7); PDR: 4 (6.3); macular edema: 2 (3.2)	NR	Medical records
Eppens et al,^12^ 2006	Australia	Prospective cohort	Median (IQR), 13.2 (11.6 to 15.0)^a^	Median (IQR), 15.3 (13.6 to 16.4)^a^	Median (IQR), 1.3 (0.6 to 3.1)^a^	1 (4.0)	25	NR	7-Field stereoscopic fundus photography	Modified Airlie House
Shield et al,^61^ 2009	Ireland; UK	Prospective cohort	Median (IQR), 13.6 (9.9 to 16.8)^a^	Median (IQR), 14.5 (10.8 to 17.8)^a^	Median, 1.0^a^	0	55	NR	NR	NR
Ruhayel et al,^62^ 2010	Australia	Prospective cohort	Mean (SD), 11.6 (1.9)^a^	Mean (SD), 16.8 (1.7)^a^	Mean (SD), 5.2 (2.0)^a^	4 (25.0)	16	NPDR: 4 (25.0); 3 with unilateral hard exudates and 1 with dot hemorrhages	NR	Medical records
Jefferies et al,^63^ 2012	New Zealand	Prospective cohort	Median (IQR), 12.9 (7.1 to 15.5)	NR	NR	0	52	NR	NR	NR
Schmidt et al,^64^ 2012	Austria; Germany	Prospective cohort	Mean (SD), 13.5 (3.4)	Mean (SD), 15.3 (3.0)	NR	12 (1.8)	684	NR	NR	NR
Jensen et al,^66^ 2021^b^	US	Prospective cohort	<20.0	NR	Mean (SD), 7.5 (2.1)	140 (31.3)	447	NR	Fundus photography (type NR)	NR
					Mean (SD), 12.4 (2.1)	126 (55.0)	229	Mild NPDR: 91 (39.7); moderate NPDR: 26 (11.4); PDR: 9 (4.0)		
Preechasuk et al,^67^ 2022	Thailand	Prospective cohort	Mean (SD), 16.9 (6.4)	Mean (SD), 23.4 (8.5)	Median (IQR), 5.2 (1.6-9.4)	8 (9.0)	89	Mild to moderate NPDR: 4 (4.5); severe NPDR: 4 (4.5)	NR	Presence of any severity of DR, macular edema, vitreous hemorrhage, or tractional retinal detachment
TODAY Study Group,^68^ 2013	US	Prospective cohort	NR	Mean (SD), 18.1 (2.5)	Mean (SD), 4.9 (1.5)	71 (13.7)	517	Minimal NPDR: 64 (12.3); mild NPDR: 7 (1.4)	7-Field stereoscopic fundus photography	ETDRS
TODAY Study Group,^65^ 2021	US	Prospective cohort	NR	Mean (SD), 25.4 (2.5)	Mean (SD), 12.0 (1.5)	210 (50.0)	420	Minimal NPDR: 95 (22.6); mild NPDR: 68 (16.2); moderate NPDR: 16 (3.8); moderately severe NPDR: 3 (0.7); severe NPDR: 5 (1.2); early or stable, treated PDR: 10 (2.4); high-risk PDR: 5 (1.2); macular edema: 14 (3.3)	7-Field stereoscopic fundus photography	ETDRS
Zuckerman Levin et al,^69^ 2022	Israel	Prospective cohort	Mean (SD), 14.7 (1.9)	Mean (SD), 14.7 (1.9)	At presentation: 0	At presentation: 4 (1.9)	At presentation: 216	NR	NR	Progressive retinal changes (nonproliferative or proliferative)
					At follow-up: mean (SD), 2.9 (2.1)	At follow-up: 5 (4.6)	At follow-up: 108			

Abbreviations: DR, diabetic retinopathy; ETDRS, Early Treatment Diabetic Retinopathy Study; NPDR, nonproliferative diabetic retinopathy; NR, not reported; PDR, proliferative diabetic retinopathy; T2D, type 2 diabetes.

^a^ Represents value for whole cohort in study, not just patients screened for retinopathy.

^b^ Abstract only.

^c^ In this abstract, there was 1 participant with Prader-Willi syndrome who was diagnosed with T2D at age 6.5 years.

All patients were diagnosed with T2D between ages 6.5 years and 21.0 years, with 1 patient diagnosed at age 6.5 years and having a background diagnosis of Prader-Willi syndrome. The diabetes duration ranged from inclusion at diabetes diagnosis to 15.0 years after diagnosis.

### Pooled Global Prevalence of DR

**Figure 1.**
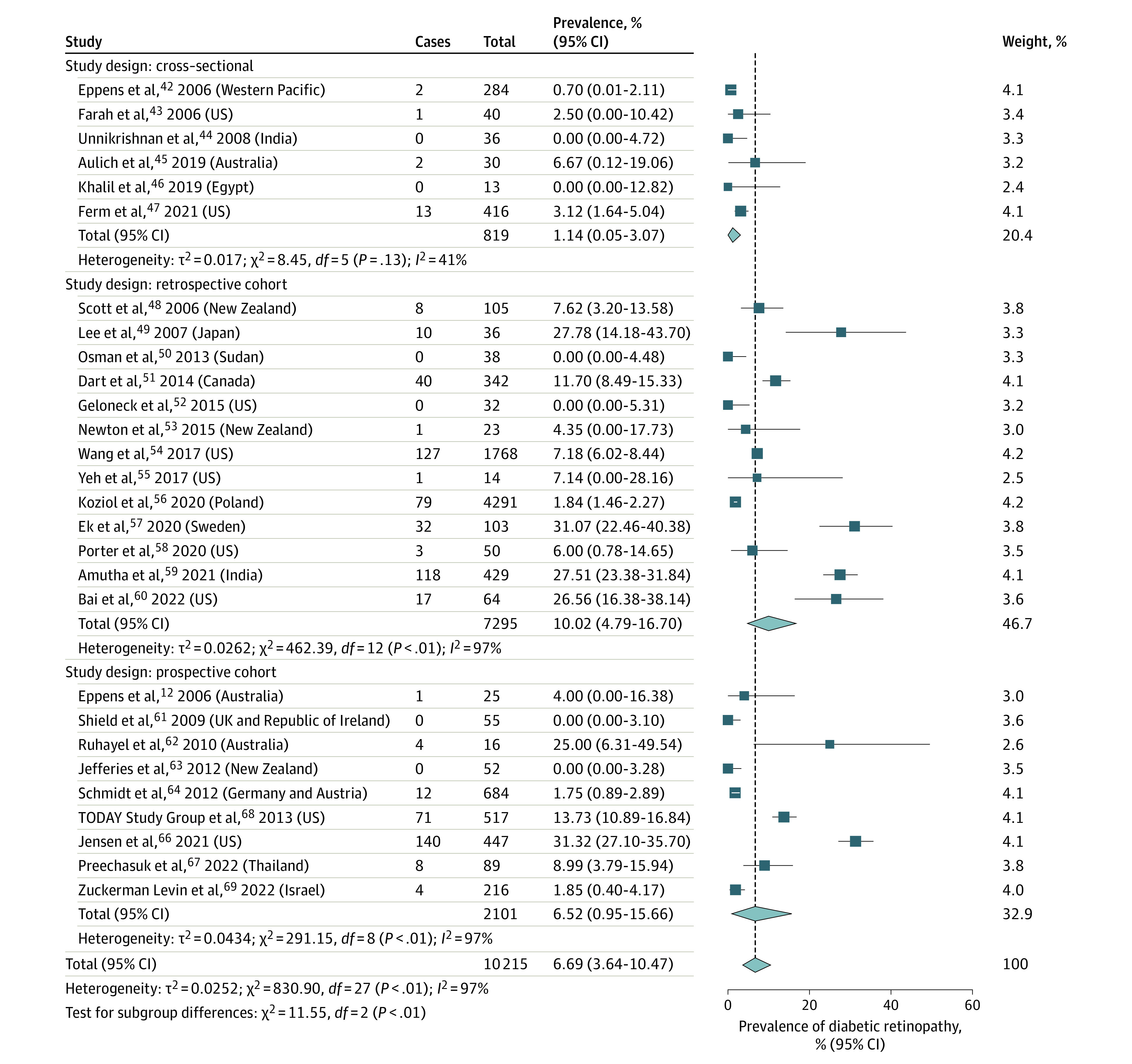
Prevalence of Diabetic Retinopathy in Youths With Type 2 Diabetes by Study Design

The number of DR cases was small, and heterogeneity was high across studies. Among 29 eligible studies,^12,42,43,44,45,46,47,48,49,50,51,52,53,54,55,56,57,58,59,60,61,62,63,64,65,66,67,68,69^ 1 study^65^ was excluded from the pooled analysis because it provided follow-up data on the same patient set included in another study,^68^ and 1 study^56^ was excluded because it provided the number of observations of DR, but it was unclear whether the data included unique patients. In this article, we report the prevalence values without and with the inclusion of the latter study^56^ because the results did not change substantially. The pooled global prevalence of DR across 27 studies^12,42,43,44,45,46,47,48,49,50,51,52,53,54,55,57,58,59,60,61,62,63,64,66,67,68,69^ involving 5924 unique patients was 6.99% (95% CI, 3.75%-11.00%; *I*^2^ = 95%; *P* < .001; 615 patients) (Figure 1). The DR prevalence was 1.14% (95% CI, 0.05%-3.07%; *I*^2^ = 41%; *P* = .13; 18 of 819 patients) in cross-sectional studies,^42,43,44,45,46,47^ 11.29% (95% CI, 5.82%-18.10%; *I*^2^ = 94%; *P* < .001; 357 of 3004 patients) in retrospective cohort studies,^48,49,50,51,52,53,54,55,57,58,59,60^ and 6.52% (95% CI, 0.95%-15.66%; *I*^2^ = 97%; *P* < .001; 240 of 2101 patients) in prospective cohort studies.^12,61,62,63,64,66,67,68,69^ When including the study that reported the number of observations of patients with T2D and DR,^56^ the pooled global prevalence was 6.69% (95% CI, 3.64%-10.47%; *I*^2^ = 97%; *P* < .001; 4291 observations; 10 215 total unique patients plus observations).

The exploratory analysis using the random intercept mixed-effects logistic regression model, in which the results were compared with the Freeman-Tukey double arcsine analysis, had consistent results with overlapping 95% CIs. All outcomes, with the exception of DR prevalence by race, had results somewhat close to each other using the 2 methods; for example, DR prevalence was 6.99% vs 5.03% when comparing the generalized linear mixed-effects method vs the Freeman-Tukey double arcsine transformation method. However, the data were limited. The 95% PIs were broad (including a range of 0.30%-50.75% in the generalized linear mixed-effects model vs 0%-33.99% in the Freeman-Tukey double arcsine transformation model), primarily due to the high heterogeneity among the studies (eTable 9 in Supplement 1).

### Prevalence of DR Based on Severity

**Figure 2.**
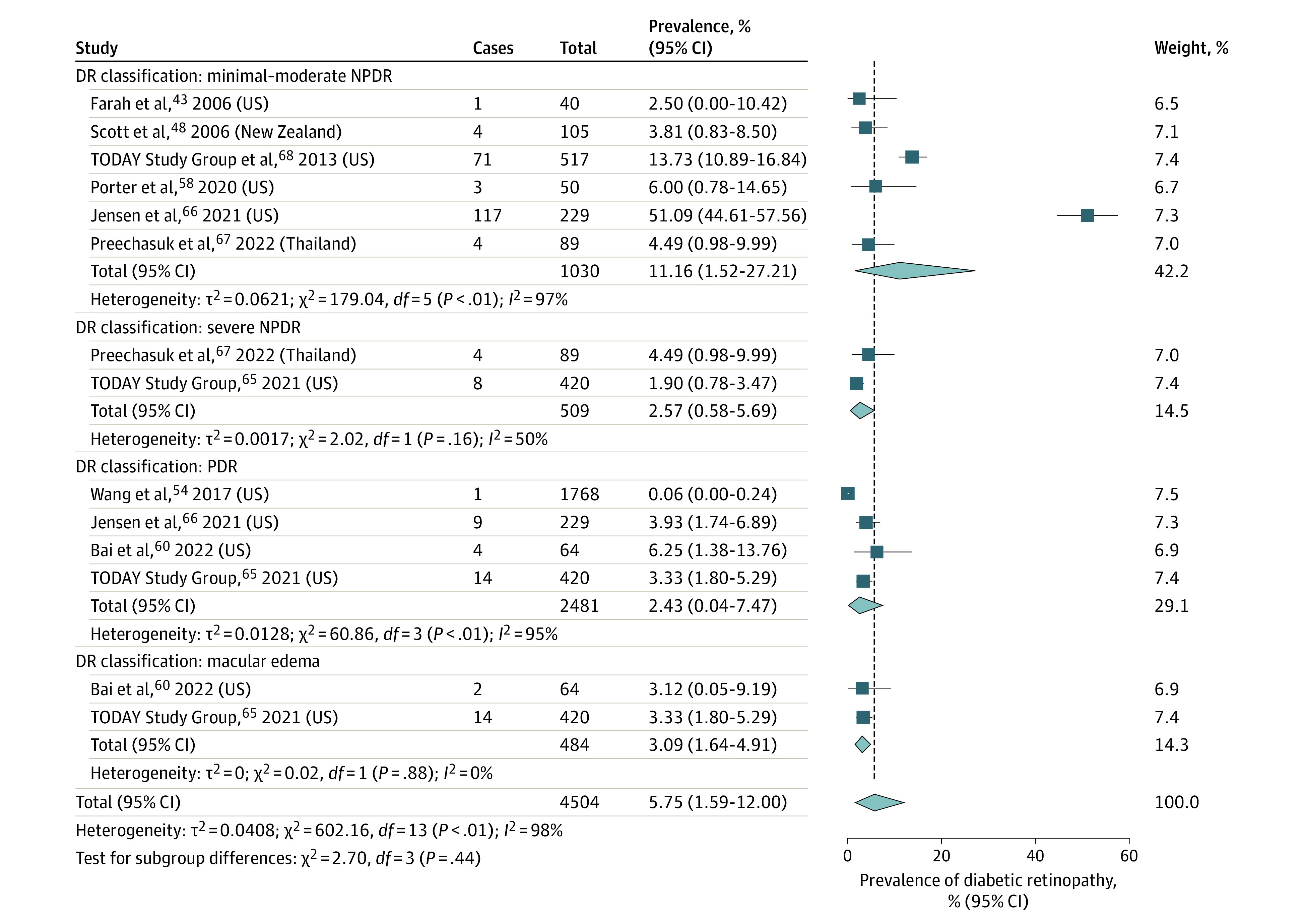
Prevalence of Diabetic Retinopathy in Youths With Type 2 Diabetes by Severity DR indicates diabetic retinopathy; NPDR, nonproliferative diabetic retinopathy; and PDR, proliferative diabetic retinopathy.

Only 9 studies^43,48,54,58,60,65,66,67,68^ reported DR classification using Early Treatment for Diabetic Retinopathy Study criteria or modified Airlie House criteria, which both classify DR severity based on the presence and extent of retinal thickening, microaneurysms, cotton wool spots, dot blot hemorrhages, venous beading, intraretinal microvascular anomalies, and neovascularization. The prevalence of minimal to moderate NPDR was 11.16% (95% CI, 1.52%-27.21%; *I*^2^ = 97%; *P* < .001; 200 of 1030 patients),^43,48,58,66,67,68^ the prevalence of severe NPDR was 2.57% (95% CI, 0.58%-5.69%; *I*^2^ = 50%; *P* = .16; 12 of 509 patients),^65,67^ the prevalence of proliferative DR was 2.43% (95% CI, 0.04%-7.47%; *I*^2^ = 95%; *P* < .001; 28 of 2481 patients),^54,60,65,66^ and the prevalence of macular edema was 3.09% (95% CI, 1.64%-4.91%; *I*^2^ = 0%; *P* = .88; 16 of 484 patients) (Figure 2).^60,65^

### Prevalence of DR Based on Method of Retinopathy Assessment

**Figure 3.**
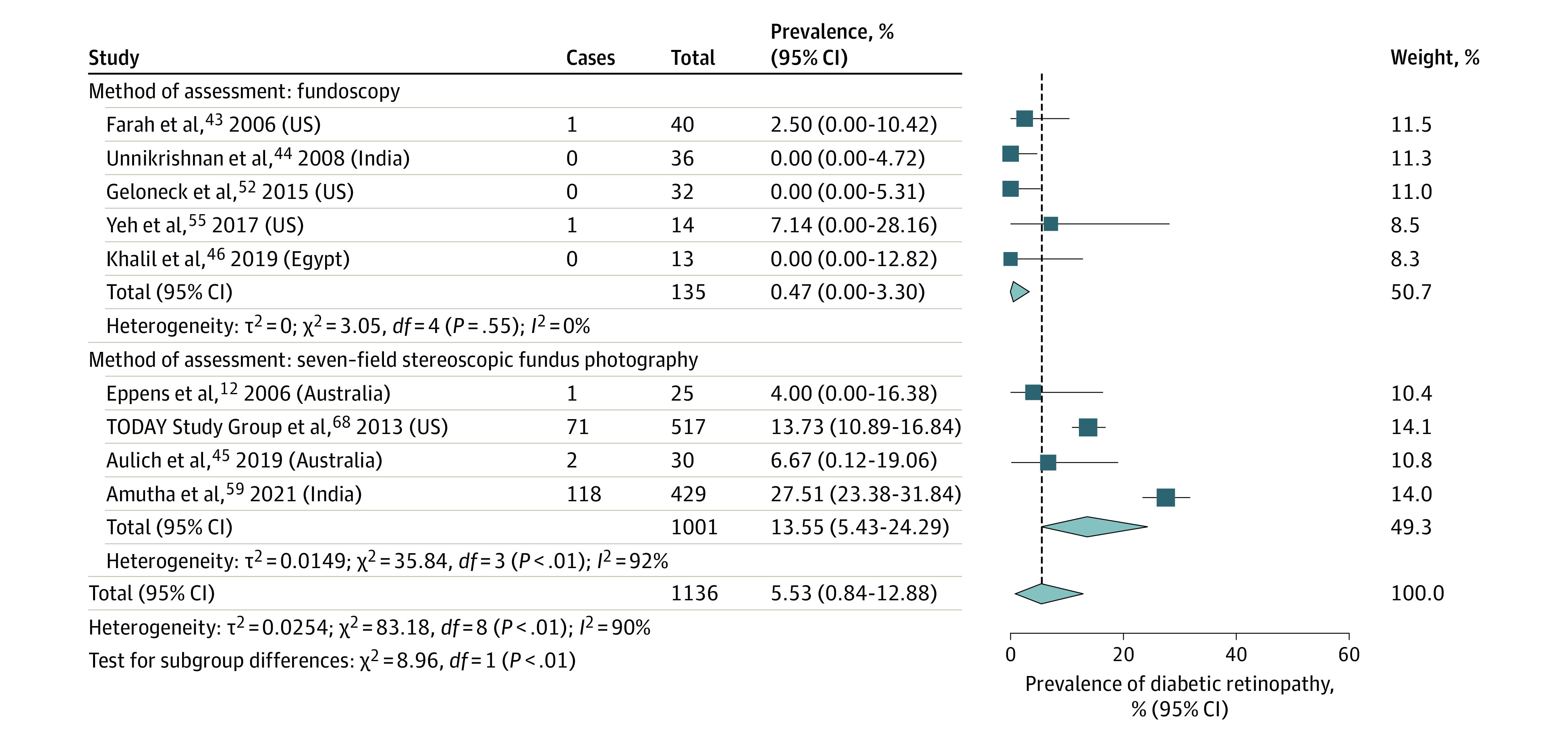
Prevalence of Diabetic Retinopathy in Youths With Type 2 Diabetes by Method of Assessment

The prevalence of DR in 5 studies^43,44,46,52,55^ using fundoscopy to diagnose DR was 0.47% (95% CI, 0%-3.30%; *I*^2^ = 0%; *P* = .55; 2 of 135 patients). The prevalence of DR in 4 studies^12,45,59,68^ using 7-field stereoscopic fundus photography to diagnose DR was 13.55% (95% CI, 5.43%-24.29%; *I*^2^ = 92%; *P* < .001; 192 of 1001 patients) (Figure 3). Other assessment methods included nonmydriatic fundus photography in 1 study,^47^ and an unspecified form of fundus photography in 3 studies.^48,57,66^ A total of 15 studies^42,49,50,51,53,54,56,58,60,61,62,63,64,67,69^ did not report the DR assessment method used.

### Global Prevalence of DR Based on Diabetes Duration

Analyzing only prospective cohort studies that reported mean T2D duration,^61,62,65,66,67,68,69^ the prevalence of DR with T2D duration of less than 2.5 years was 1.11% (95% CI, 0.04%-3.06%; *I*^2^ = 5%; *P* = .30; 4 of 271 patients)^61,69^ (eFigure 2 in Supplement 1). For T2D duration of 2.5 to 5.0 years, the prevalence was 9.04% (95% CI, 2.24%-19.55%; *I*^2^ = 88%; *P* < .001; 76 of 625 patients)^68,69^; for T2D duration of greater than 5.0 years, the prevalence was 28.14% (95% CI, 12.84%-46.45%; *I*^2^ = 96%; *P* < .001; 362 of 972 patients).^62,65,66,67^ When analyzing only retrospective cohort studies that reported mean T2D duration,^48,52,53,58^ DR prevalence was similar for T2D duration of less than 2.5 years (5.30%; 95% CI, 0.87%-12.19%; *I*^2^ = 0%; *P* = .90; 4 of 73 patients)^53,58^ and T2D duration of 2.5 to 5.0 years (3.09%; 95% CI, 0%-14.06%; *I*^2^ = 75%; *P* = .05; 8 of 137 patients)^48,52^ (eFigure 3 in Supplement 1). Analyzing only cross-sectional studies^42,43,44,45,46,47^ was not possible because these studies did not report all ranges of T2D duration. When all study designs^12,42,43,44,45,46,47,48,49,50,51,52,53,54,55,56,57,58,59,60,61,62,63,64,65,66,67,68,69^ were included in the analysis, the prevalence of DR at less than 2.5 years after diagnosis of diabetes was 1.78% (95% CI, 0.25%-4.20%; *I*^2^ = 23%; *P* = .26; 9 of 384 patients).^43,53,58,61,69^ The prevalence increased sharply at 2.5 to 5.0 years after diagnosis to 5.08% (95% CI, 1.04%-11.22%; *I*^2^ = 80%; *P* < .001; 84 of 775 patients),^46,48,52,68,69^ with a further increase to 28.83% (95% CI, 15.97%-43.63%; *I*^2^ = 95%; *P* < .001; 394 of 1075 patients)^57,62,65,66,67^ at 5.0 years after diagnosis (eFigure 4 in Supplement 1).

### Global Prevalence of DR Based on Sex

The odds ratio of DR prevalence was lower in male vs female patients (0.40; 95% CI, 0.02-7.21; *I*^2^ = 75%; *P* = .53; 45 of 88 male patients vs 91 of 177 female patients)^49,66^ (eFigure 5 in Supplement 1), with a wide 95% CI that prevented reaching a definite conclusion on sex differences in DR prevalence. Two studies^54,57^ did not report a sex-specific prevalence value but found that male patients had a higher risk of DR than female patients when examining hazard ratios.

### Prevalence of DR Based on Race

Racial classifications were based on medical records^48,50,52,53,60,69^ or self-reporting^60,63,66^ by participants in the studies. The overall pooled prevalence of DR in Middle Eastern or White patients was 24.07% (95% CI, 6.26%-47.91%; *I*^2^ = 88%; *P* < .001; 57 of 171 patients).^46,57,66^ Asian patients had a prevalence of 13.31% (95% CI, 2.49%-30.05%; *I*^2^ = 93%; *P* < .001; 136 of 590 patients)^44,49,59,67^ (eFigure 6 in Supplement 1). There were insufficient data to assess the pooled prevalence in other racial groups.

### Association of Age, Diabetes Duration, Obesity, HbA_1c_ Level, and Hypertension With DR Prevalence

Age (*P* < .001; 15 patients), diabetes duration (*P* = .02; 13 patients), and hypertension prevalence (*P* = .03; 17 patients) were positively associated with DR prevalence. Meta-regression analysis revealed no associations between obesity prevalence (*P* = .93; 13 patients) or mean age at diabetes diagnosis (*P* = .26; 14 patients) and DR prevalence. In addition, there was no association between glycemic control (*P* = .60; 18 patients) and DR prevalence after examining the data in aggregate and by study design. However, patients with T2D who developed DR had a higher HbA_1c_ level when compared with patients without retinopathy (mean HbA_1c_ difference, 1.37 (95% CI, 0.95-1.79; *I*^2^ = 0%; *P* < .001)^47,68^ (eFigure 7 in Supplement 1).

### Risk of Bias, Level of Evidence, and Publication Bias

Most studies had a low risk of bias (8 studies^44,47,52,54,56,57,58,60^ [27.6%]) or a moderate risk of bias (20 studies^12,42,43,45,46,48,50,51,53,55,59,61,62,63,64,65,66,67,68,69^ [69.0%]), with only 1 study^49^ (3.4%) having a high risk of bias (eTable 10 in Supplement 1). The risk of bias was higher if the patients were from a single clinic or city and not a nationally representative sample; data from those studies^12,43,45,46,48,49,50,51,52,53,55,58,59,60,62,63,67^ may therefore have limited generalizability. Some studies^42,43,49^ did not use representative sampling frameworks, and others^43,45,49,65,66,68^ did not take a census or randomly select patients. Some studies^12,46,53,55,59,62,64,65,66,68,69^ also had missing data of greater than 25%, potentially leading to nonresponse bias. 

The risk of bias was also present if the definition of DR or the assessment method was not described, which occurred in 7 studies.^42,49,61,63,64,67,69^ In some studies,^44,48,54,56,61,64^ it was unclear whether all participants were examined using the same methods. A total of 14 studies^12,47,51,54,56,57,58,59,60,61,63,64,67,69^ (48.3%) had the highest level of evidence (level 1), while 9 studies^42,44,46,48,50,52,53,55,62^ (31.0%) had level 2 evidence, and 6 studies^43,45,49,65,66,68^ (20.7%) had level 3 evidence. No publication bias was identified for the prevalence of DR outcome (eFigure 8 in Supplement 1) based on the Egger test (*P* = .52).

### Sensitivity Analysis

Results of the sensitivity analyses are shown in eTable 11 in Supplement 1. Of note, excluding the studies that involved patients older than 18 years decreased the pooled estimate of DR prevalence to 3.03% (95% CI, 1.02%-5.81%) (Figure 1; eTable 11 in Supplement 1), suggesting that DR risk increased with age.

## Discussion

This systematic review and meta-analysis found that the global prevalence of DR was 6.99% among children with T2D, and DR prevalence increased significantly at more than 5 years after T2D diagnosis. The current data suggest that approximately 1 in 14 children and adolescents with T2D will have DR within a few years after diabetes diagnosis. While most patients included in this review had minimal or mild NPDR, a substantial minority had more severe disease, such as proliferative DR or macular edema, that can lead to visual impairment and potentially irreversible vision loss. Notably, there was some evidence to suggest that the prevalence of DR rapidly increased with age and diabetes duration; almost 1 in 4 children with T2D for 5 years or more developed DR. The analysis of the associations of sex and race with DR prevalence was inconclusive due to limited data. The scale of DR prevalence in youths with T2D supports the recommendations for periodic patient screening.^21,22^

When the data from pediatric patients with T2D are compared with those of pediatric patients with type 1 diabetes (T1D), only 2% of children with T1D develop mild NPDR, irrespective of their age at diabetes onset, and none develop proliferative DR or macular edema.^70^ However, DR prevalence increases sharply at 5 years after diagnosis, to approximately 25%.^70^ Clinical practice guidelines for T1D currently recommend screening for DR at puberty or beginning at age 11 years if the child has had diabetes for 2 to 5 years.^71^ The early years of transition from pediatric to adult care among patients aged 18 to 21 years are also associated with an increase in DR among patients with T1D.^72^ Surveillance for DR is important during this transitional stage, when many patients also have difficulty maintaining glycemic control.^72^

Among adults with T2D, 21% to 39% of patients have DR at diagnosis, and the rates increase thereafter.^19,73^ Our results suggested that diabetes duration was positively associated with DR in pediatric T2D. Notably, the findings of the current review suggest that this increase is emerging decades earlier among these children compared with adults with T2D. The long-term outcomes of DR are not yet known due to the relative novelty of the condition. Longitudinal studies are warranted to assess these outcomes.

Hyperglycemia can result in structural and functional retinal abnormalities in pediatric patients with T2D as early as 2 years after diagnosis.^74^ Adolescents with T2D have substantial multifocal electroretinographic implicit time delays compared with youths with T1D and youths without diabetes.^74^ In adults with T2D, similar findings have suggested impairment of neural retinal function, future vascular lesions, and increased risk of DR.^74,75,76,77,78^ In addition, adolescents with T2D had substantially lower retinal thickness and retinal venular dilation when compared with patients without diabetes.^74^ These findings suggest that retinal abnormalities are present early in pediatric T2D, so it is important that screening be undertaken to detect DR early in this population to prevent impaired vision and blindness.

While the current pediatric T2D clinical practice guidelines recommend regular screening of DR at baseline and annually thereafter, screening guidelines are not routinely followed.^21,22^ Only 22% to 54% of pediatric patients with T2D have had dilated eye examinations.^79,80^ Because the findings of this review suggest the prevalence of DR increases rapidly with diabetes duration, there is an immediate need for regular screening to be performed consistently. The benefits of early identification of DR include increased focus on improving glycemic control to minimize microvascular disease, maintaining blood pressure, and streamlining the monitoring of DR progression.^19,54^ Intensive glycemic control in adolescents with T1D reduced DR by 53% in the Diabetes Control and Complications Trial.^81,82^ Similarly, the UK Prospective Diabetes Study in adults with T2D showed that strict glycemic and blood pressure control reduced DR progression by 34%.^83,84^ In our study, hypertension prevalence was associated with DR prevalence, suggesting that hypertension may also be associated with DR in pediatric T2D. However, although patients with higher HbA_1c_ values had higher DR prevalence, this finding did not reach statistical significance. The analysis of the association of DR with HbA_1c_ levels yielded variable results, with some studies^47,49,54,57,62,65,68^ reporting an association and other studies^52,58,67^ finding no association. When pooling studies that reported mean HbA_1c_ values in patients with and without DR,^47,68^ the mean difference suggested significantly higher HbA_1c_ levels in patients with vs without DR. It is possible that our analysis of mean HbA_1c_ levels did not reveal an association with DR prevalence because it was based on mean HbA_1c_ values extracted from cross-sectional data, which may not account for longitudinal fluctuations in glycemic control over time. In addition, there is some evidence to suggest that there are racial and ethnic differences in HbA_1c_ levels, with Asian, Black, Hispanic, and Indigenous individuals having higher HbA_1c_ values than White individuals.^85^ While maintaining adequate glycemic control is an important step in preventing microvascular complications, any association between glycemic control and DR is likely polygenic.^19^

The screening method had implications for DR prevalence and explained some of the heterogeneity among studies included in this review, and 7-field stereoscopic fundus photography identified more cases of DR than fundoscopy. Compared with the gold standard of 7-field stereoscopic photography, indirect fundoscopy has a sensitivity of 76% and a specificity of 95%.^86^ In contrast, 4-field wide-angle stereoscopic photography has a sensitivity of 94% and a specificity of 96%.^86^ Digital nonmydriatic imaging has a sensitivity of 98% and a specificity of 86% for detecting DR, and direct fundoscopy with pupil dilation has a sensitivity of 65% and a specificity of 97%.^86^ It is important to note that all but 1^43^ of the included studies^12,42,43,44,45,46,47,48,49,50,51,52,53,54,55,57,58,59,60,61,62,63,64,65,66,67,68,69^ did not specify whether direct or indirect fundoscopy was performed. Fundus photography is more sensitive than fundoscopy for diagnosing mild cases of DR, while fundoscopy is better at detecting retinal thickening from macular edema and early neovascularization.^19,52,87^ Although both methods are accepted in screening guidelines, it has been suggested that fundus photography be used in the pediatric population because fundoscopy is challenging to perform in children.^52^ Fundus photography requires specialized equipment and trained staff to acquire the images and perform the analysis.^19,88^ These technologies may not be accessible, especially in low- and middle-income countries where pediatric T2D rates are rapidly increasing.^89^ The disparity in global access to health care resources impacts adherence to and generalizability of screening guidelines, and advocacy for access to retinal imaging and training personnel in different health care settings is important.^90^ Furthermore, emerging technologies, such as low-cost cameras, automated grading of retinal images, and virtual access to specialist assessments and care, will likely offer more equitable access to care and improve outcomes.^91,92^ For example, autonomous artificial intelligence systems have been developed for the early detection of DR. These systems have been reported to have a sensitivity of 85.7% and a specificity of 79.3% in diagnosing more than mild severity of DR in youths when compared with consensus grading by retinal specialists.^93,94^ While these systems are not yet approved for use in children, they offer a promising screening solution because they do not require supervision by eye care professionals and can be used in settings in which specialists are not available.^93^

Data on DR prevalence in pediatric T2D by sex or race were scarce. No conclusions about DR prevalence by sex or race could be reached due to the limited data. National estimates of DR among US and UK adults suggest that Black, Hispanic, and South Asian individuals have a 5% to 10% higher prevalence of DR compared with White individuals.^95,96,97^ In the SEARCH for Diabetes in Youth Study,^98^ youths with T2D from non-White racial groups had a higher prevalence of DR (odds ratio, 2.05; 95% CI, 0.97-4.33). Differences in HbA_1c_ level or diabetes duration did not explain this finding.^98^ An extensive study of ophthalmic screening patterns in the US^80^ found that Black and Hispanic children, especially those with low socioeconomic status, had 11% to 18% lower rates of DR screening than White children. These findings highlight the need for the creation of equitable screening strategies for DR that can reach all pediatric patients with T2D.

### Limitations

This study has several limitations. The heterogeneity was high across studies. Some studies did not report the DR assessment method^42,49,50,51,53,54,56,58,60,61,62,63,64,67,69^ or the type of fundus photography^48,57,66^ used. Data on DR prevalence by sex and race were limited, so we could not reach any conclusions.

In addition, the small number of DR cases in the included studies can have implications for the reliability of the variance and pooled estimates.^34^ For this reason, we used the Freeman-Tukey double arcsine transformation^99,100^ to stabilize variance in our meta-analysis. We also conducted a generalized linear mixed analysis for each outcome to assess the robustness of the results^99,100^ (eTable 11 in Supplement 1). We ultimately presented the results of our Freeman-Tukey transformation analysis because generalized linear mixed models do not provide a weight for each study, which is important information for clinicians to have access to when making practice recommendations.^99,100^ We calculated 95% PIs alongside 95% CIs to provide information on the estimated range of true case rates in this study (eTable 11 in Supplement 1). Given the high heterogeneity across studies included in this review, the 95% PIs were broad for some outcomes and suggested the need for more high-quality, adequately powered longitudinal studies.

## Conclusions

The findings of this systematic review and meta-analysis suggest that the retinal microvasculature is an early target of T2D in children and that the risk of DR continues to increase over time. Mechanistic insights into the pathogenesis of DR in children with T2D remain limited, and this area warrants prioritized investigation. Increasing the number of children with T2D who undergo regular DR screening is important to meet current clinical practice guideline standards. Fundus photography is more sensitive in diagnosing early DR than fundoscopy. These assessments will likely maintain vision and quality of life and improve long-term outcomes. Equitable access to health care resources to detect and treat DR is a global priority that needs increased attention.

## Notes

Supplement 1. **eTable 1.** MOOSE Checklist **eTable 2.** Search Strategy—MEDLINE **eTable 3.** Search Strategy—Embase **eTable 4.** Search Strategy—CINAHL **eTable 5.** Search Strategy—Cochrane Library: Cochrane Central Register of Controlled Trials and Cochrane Database of Systematic Reviews **eTable 6.** Search Strategy—Web of Science: Conference Proceedings Citation Index–Science **eTable 7.** Type 2 Diabetes Criteria Used Across the Included Studies **eTable 8.** Characteristics of Included Studies **eTable 9.** Comparison of Meta-analysis Results Using Freeman-Tukey Double Arcsine Transformation and Generalized Linear Mixed-Effects Model With Confidence and Prediction Intervals **eTable 10.** Risk of Bias of Included Studies **eTable 11.** Results of Sensitivity Analysis **eFigure 1.** PRISMA Flow Diagram Illustrating Study Selection **eFigure 2.** Forest Plot of Prevalence of Diabetic Retinopathy in Pediatric Type 2 Diabetes by Diabetes Duration in Prospective Cohort Studies **eFigure 3.** Forest Plot of Prevalence of Diabetic Retinopathy in Pediatric Type 2 Diabetes by Diabetes Duration in Retrospective Cohort Studies **eFigure 4.** Forest Plot of Prevalence of Diabetic Retinopathy in Pediatric Type 2 Diabetes by Diabetes Duration in All Studies **eFigure 5.** Forest Plot of the Odds Ratio of Diabetic Retinopathy in Pediatric Type 2 Diabetes by Sex **eFigure 6.** Forest Plot Illustrating Prevalence of Diabetic Retinopathy in Pediatric Type 2 Diabetes by Race **eFigure 7.** Forest Plot Showing Mean Difference in HbA_1c_ in Participants With vs Without Diabetic Retinopathy **eFigure 8.** Funnel Plot Examining Publication Bias for Diabetic Retinopathy Prevalence Outcome
eReferences.

Supplement 2.
Data Sharing Statement
