# Supplementary material for: Global Prevalence of Diabetic Retinopathy in Pediatric Type 2 Diabetes: A Systematic Review and Meta-analysis
Source: JAMA Netw Open. 2023 Mar 17;6(3):e231887. doi: 10.1001/jamanetworkopen.2023.1887 (PMC10024209; doi:10.1001/jamanetworkopen.2023.1887)
Supplement: Supplement 1. — eTable 1. MOOSE Checklist eTable 2. Search Strategy—MEDLINE eTable 3. Search Strategy—Embase eTable 4. Search Strategy—CINAHL eTable 5. Search Strategy—Cochrane Library: Cochrane Central Register of Controlled Trials and Cochrane Database of Systematic Reviews eTable 6. Search Strategy—Web of Science: Conference Proceedings Citation Index–Science eTable 7. Type 2 Diabetes Criteria Used Across the Included Studies eTable 8. Characteristics of Included Studies eTable 9. Comparison of Meta-analysis Results Using Freeman-Tukey Double Arcsine Transformation and Generalized Linear Mixed-Effects Model With Confidence and Prediction Intervals eTable 10. Risk of Bias of Included Studies eTable 11. Results of Sensitivity Analysis eFigure 1. PRISMA Flow Diagram Illustrating Study Selection eFigure 2. Forest Plot of Prevalence of Diabetic Retinopathy in Pediatric Type 2 Diabetes by Diabetes Duration in Prospective Cohort Studies eFigure 3. Forest Plot of Prevalence of Diabetic Retinopathy in Pediatric Type 2 Diabetes by Diabetes Duration in Retrospective Cohort Studies eFigure 4. Forest Plot of Prevalence of Diabetic Retinopathy in Pediatric Type 2 Diabetes by Diabetes Duration in All Studies eFigure 5. Forest Plot of the Odds Ratio of Diabetic Retinopathy in Pediatric Type 2 Diabetes by Sex eFigure 6. Forest Plot Illustrating Prevalence of Diabetic Retinopathy in Pediatric Type 2 Diabetes by Race eFigure 7. Forest Plot Showing Mean Difference in HbA1c in Participants With vs Without Diabetic Retinopathy eFigure 8. Funnel Plot Examining Publication Bias for Diabetic Retinopathy Prevalence Outcome eReferences. [file jamanetwopen-e231887-s001.pdf]

Supplementary Online Content

Cioana M, Deng J, Nadarajah A, et al. Global prevalence of diabetic retinopathy in pediatric type 2 diabetes: a systematic review and meta-analysis. *JAMA Netw Open*. 2023;6(3):e231887. doi:10.1001/jamanetworkopen.2023.1887

eTable 1. MOOSE Checklist

eTable 2. Search Strategy—MEDLINE

eTable 3. Search Strategy—Embase

eTable 4. Search Strategy—CINAHL

eTable 5. Search Strategy—Cochrane Library: Cochrane Central Register of Controlled Trials and Cochrane Database of Systematic Reviews

eTable 6. Search Strategy—Web of Science: Conference Proceedings Citation Index–Science

eTable 7. Type 2 Diabetes Criteria Used Across the Included Studies

eTable 8. Characteristics of Included Studies

eTable 9. Comparison of Meta-analysis Results Using Freeman-Tukey Double Arcsine Transformation and Generalized Linear Mixed-Effects Model With Confidence and Prediction Intervals

eTable 10. Risk of Bias of Included Studies

eTable 11. Results of Sensitivity Analysis

eFigure 1. PRISMA Flow Diagram Illustrating Study Selection

eFigure 2. Forest Plot of Prevalence of Diabetic Retinopathy in Pediatric Type 2 Diabetes by Diabetes Duration in Prospective Cohort Studies

eFigure 3. Forest Plot of Prevalence of Diabetic Retinopathy in Pediatric Type 2 Diabetes by Diabetes Duration in Retrospective Cohort Studies

eFigure 4. Forest Plot of Prevalence of Diabetic Retinopathy in Pediatric Type 2 Diabetes by Diabetes Duration in All Studies

eFigure 5. Forest Plot of the Odds Ratio of Diabetic Retinopathy in Pediatric Type 2 Diabetes by Sex

eFigure 6. Forest Plot Illustrating Prevalence of Diabetic Retinopathy in Pediatric Type 2 Diabetes by Race

eFigure 7. Forest Plot Showing Mean Difference in HbA_{1c} in Participants With vs Without Diabetic Retinopathy

eFigure 8. Funnel Plot Examining Publication Bias for Diabetic Retinopathy Prevalence Outcome

eReferences

This supplemental material has been provided by the authors to give readers additional information about their work.

© 2023 Cioana M et al. *JAMA Network Open*.

eTable 1. MOOSE Checklist[1]

Item No	Recommendation	Reported on Page No
Reporting of background should include		
1	Problem definition	5
2	Hypothesis statement	N/A
3	Description of study outcome(s)	5
4	Type of exposure or intervention used	5
5	Type of study designs used	5-7
6	Study population	5-7
Reporting of search strategy should include		
7	Qualifications of searchers (eg, librarians and investigators)	6
8	Search strategy, including time period included in the synthesis and key words	eTables 2-6
9	Effort to include all available studies, including contact with authors	6-7
10	Databases and registries searched	6
11	Search software used, name and version, including special features used (eg, explosion)	6-8
12	Use of hand searching (eg, reference lists of obtained articles)	6-7
13	List of citations located and those excluded, including justification	6-7
14	Method of addressing articles published in languages other than English	6-7
15	Method of handling abstracts and unpublished studies	6-7
16	Description of any contact with authors	6-7
Reporting of methods should include		
17	Description of relevance or appropriateness of studies assembled for assessing the hypothesis to be tested	6-8
18	Rationale for the selection and coding of data (eg, sound clinical principles or convenience)	6-8
19	Documentation of how data were classified and coded (eg, multiple raters, blinding and interrater reliability)	7
20	Assessment of confounding (eg, comparability of cases and controls in studies where appropriate)	7-8
21	Assessment of study quality, including blinding of quality assessors, stratification or regression on possible predictors of study results	7
22	Assessment of heterogeneity	6-8
23	Description of statistical methods (eg, complete description of fixed or random effects models, justification of whether the chosen models account for predictors of study results, dose-response models, or cumulative meta-analysis) in sufficient detail to be replicated	7-8
24	Provision of appropriate tables and graphics	Table 1, Figures 1-4
Reporting of results should include		

© 2023 Cioana M et al. *JAMA Network Open*.

25	Graphic summarizing individual study estimates and overall estimate	Figure 1
26	Table giving descriptive information for each study included	Table 1
27	Results of sensitivity testing (eg, subgroup analysis)	8-12, eTable 9
28	Indication of statistical uncertainty of findings	8-12
Reporting of discussion should include		
29	Quantitative assessment of bias (eg, publication bias)	11-12
30	Justification for exclusion (eg, exclusion of non-English language citations)	7
31	Assessment of quality of included studies	11-12
Reporting of conclusions should include		
32	Consideration of alternative explanations for observed results	12-17
33	Generalization of the conclusions (ie, appropriate for the data presented and within the domain of the literature review)	12-17
34	Guidelines for future research	12-17
35	Disclosure of funding source	18

© 2023 Cioana M et al. *JAMA Network Open*.

eTable 2. Search Strategy—MEDLINE

1	exp Diabetes Mellitus, Type 2/
2	NIDDM.ti,ab,kf.
3	MODY.ti,ab,kf.
4	t2d*.ti,ab,kf.
5	((typ* two or typ?two or typ* 2 or typ* II or typ?2 or typ?II or typ* ii or typ?ii) adj4 diabet*).ti,ab,kf.
6	((non insulin or noninsulin or late or adult* or matur* or slow or stabl*) adj4 diabet*).ti,ab,kf.
7	((ketoresist* or keto* resist* or keto* prone) adj4 diabet*).ti,ab,kf.
8	or/1-7
9	exp Child/
10	child*.ti,ab,kf.
11	adolescen*.ti,ab,kf.
12	exp Adolescent/
13	youth*.ti,ab,kf.
14	teenage*.ti,ab,kf.
15	preadolescen*.ti,ab,kf.
16	Pediatrics/
17	p?ediatric*.ti,ab,kf.
18	pe?diatric*.ti,ab,kf.
19	or/9-18
20	8 and 19
21	Diabetic Retinopathy/
22	retinopath*.ti,ab,kf.
23	retinitis.ti,ab,kf.
24	NPDR.ti,ab,kf.
25	PDR.ti,ab,kf.
26	(vitre* adj2 (detach* or hemorrhag*)).ti,ab,kf.
27	(retina* adj2 detach*).ti,ab,kf.
28	(diabet* adj3 maculopath*).ti,ab,kf.
29	or/21-28
30	20 and 29
31	Prevalence/
32	prevalence.ti,ab,kf.
33	prevalence studies/
34	Incidence/
35	incidence studies/
36	incidence.ti,ab,kf.
37	Epidemiology/

© 2023 Cioana M et al. *JAMA Network Open*.

38	epidemiolog*.ti,ab,kf.
39	ep.fs.
40	epidemiologic methods/ or epidemiological monitoring/ or sentinel surveillance/
41	exp epidemiologic studies/
42	case-control.ti,ab,kf.
43	cohort.ti,ab,kf.
44	prospective.ti,ab,kf.
45	longitudinal.ti,ab,kf.
46	retrospective.ti,ab,kf.
47	cross sectional.ti,ab,kf.
48	correlational.ti,ab,kf.
49	or/31-48
50	30 and 49
51	50 not (animals/ not (humans/ and animals/))
52	remove duplicates from 51

© 2023 Cioana M et al. *JAMA Network Open*.

eTable 3. Search Strategy—Embase

1	non insulin dependent diabetes mellitus/
2	NIDDM.ti,ab,kw.
3	MODY.ti,ab,kw.
4	t2d*.ti,ab,kw.
5	((typ* two or typ?two or typ* 2 or typ* II or typ?2 or typ?II or typ* ii or typ?ii) adj4 diabet*).ti,ab,kw.
6	((non insulin or noninsulin or late or adult* or matur* or slow or stabl*) adj4 diabet*).ti,ab,kw.
7	((ketoresist* or keto* resist*) adj6 diabet*).ti,ab,kw.
8	or/1-7
9	exp child/
10	child*.ti,ab,kw.
11	adolescent/
12	adolescen*.ti,ab,kw.
13	youth*.ti,ab,kw.
14	teenage*.ti,ab,kw.
15	preadolescen*.ti,ab,kw.
16	pediatrics/
17	p?ediatric*.ti,ab,kw.
18	pe?diatric*.ti,ab,kw.
19	or/9-18
20	8 and 19
21	diabetic retinopathy/
22	retinopath*.ti,ab,kw.
23	retinitis.ti,ab,kw.
24	NPDR.ti,ab,kw.
25	PDR.ti,ab,kw.
26	(vitre* adj2 (detach* or hemorrhag*)).ti,ab,kw.
27	(retina* adj2 detach*).ti,ab,kw.
28	(diabet* adj3 maculopath*).ti,ab,kw.
29	or/21-28
30	20 and 29
31	prevalence/
32	prevalence.ti,ab,kw.
33	incidence/
34	incidence.ti,ab,kw.
35	epidemiology/
36	epidemiolog*.ti,ab,kw.
37	ep.fs.

© 2023 Cioana M et al. *JAMA Network Open*.

38	epidemiological monitoring/
39	sentinel surveillance/
40	case-control.ti,ab,kw.
41	cohort.ti,ab,kw.
42	prospective.ti,ab,kw.
43	longitudinal.ti,ab,kw.
44	retrospective.ti,ab,kw.
45	cross sectional.ti,ab,kw.
46	correlational.ti,ab,kw.
47	or/31-46
48	30 and 47
49	48 not (animals/ not (humans/ and animals/))
50	remove duplicates from 49

© 2023 Cioana M et al. *JAMA Network Open*.

eTable 4. Search Strategy—CINAHL

#	**Query**
S1	(MH "Child+")
S2	"child*"
S3	(MH "Adolescence+")
S4	"youth*"
S5	"teenage*"
S6	(MH "Pediatrics")
S7	"p?ediatric*"
S8	"p#ediatric*"
S9	"pe#diatric*"
S10	"pediatric*"
S11	"preadolescen*"
S12	S1 OR S2 OR S3 OR S4 OR S5 OR S6 OR S7 OR S8 OR S9 OR S10 OR S11
S13	(MH "Diabetes Mellitus, Type 2")
S14	"NIDDM"
S15	"MODY"
S16	"T2D*"
S17	(typ* two or typ?two or typ* 2 or typ* II or typ?2 or typ?II) N4 diabet*
S18	(non insulin or noninsulin or late or adult* or matur* or slow or stabl*) N4 diabet*
S19	(ketoresist* or keto* resist* or keto* prone) adj4 diabet*
S20	S13 OR S14 OR S15 OR S16 OR S17 OR S18 OR S19
S21	S12 AND S20
S22	"diabet* N3 maculopath*"
S23	"retina* N2 detach*"
S24	"vitre* N2 (detach* or hemorrhag*)"
S25	"PDR"
S26	"NPDR"
S27	""retinitis""
S28	""retinopath*""
S29	(MH "Diabetic retinopathy")
S30	S22 OR S23 OR S24 OR S25 OR S26 OR S27 OR S28 OR S29
S31	(MH "Prevalence")
S32	"prevalence"

© 2023 Cioana M et al. *JAMA Network Open*.

S33	(MH "Cross Sectional Studies")
S34	"cross section*"
S35	(MH "Incidence")
S36	"incidence"
S37	(MH "Epidemiology")
S38	"epidemiolog*"
S39	(MH "Epidemiological Research")
S40	(MH "Prospective Studies") OR (MH "Cross Sectional Studies") OR (MH "Case Control Studies") OR (MH "Correlational Studies")
S41	"case control" or "cohort" or "prospective" or "retrospective" or "longitudinal" or "correlational"
S42	S31 OR S32 OR S33 OR S34 OR S35 OR S36 OR S37 OR S38 OR S39 OR S40 OR S41
S43	(S21 AND S30 AND S42) NOT (MH "Animals")

© 2023 Cioana M et al. *JAMA Network Open*.

eTable 5. Search Strategy—Cochrane Library: Cochrane Central Register of Controlled Trials and Cochrane Database of Systematic Reviews

child* OR youth* OR teenage* OR adolescen* OR pediatric* OR preadolescen* OR p?ediatric* OR pe?diatric* in Title Abstract Keyword	**AND**
NIDDM OR MODY OR t2d OR typ* two NEAR/4 diabet* OR typ?two NEAR/4 diabet* OR typ* 2 NEAR/4 diabet* OR typ* II NEAR/4 diabet* OR typ?2 NEAR/4 diabet* OR typ?II NEAR/4 diabet* OR typ* ii NEAR/4 diabet* OR typ?ii NEAR/4 diabet* OR non insulin NEAR/4 diabet* OR noninsulin NEAR/4 diabet* OR late or adult* NEAR/4 diabet* OR matur* NEAR/4 diabet* OR slow NEAR/4 diabet* OR stabl* NEAR/4 diabet* OR ketoresist* NEAR/4 diabet* OR keto* resist* NEAR/4 diabet* OR keto* prone NEAR/4 diabet* in Title Abstract Keyword	**AND**
retinopath* OR retinitis OR NPDR OR PDR OR vitre* NEAR/2 detach* OR vitre* NEAR/2 hemorrhag* retina* NEAR/2 detach* OR diabet* NEAR/3 maculopath* in Title Abstract Keyword	
(Word variations have been searched)	

© 2023 Cioana M et al. *JAMA Network Open*.

eTable 6. Search Strategy—Web of Science: Conference Proceedings Citation Index–Science

#1	TI=(child* OR youth* OR teenage* OR adolescen* OR pediatric* OR preadolescen* OR p?ediatric* OR pe?diatric*)
#2	TS=(child* OR youth* OR teenage* OR adolescen* OR pediatric* OR preadolescen* OR p?ediatric* OR pe?diatric*)
#3	#1 OR #2
#4	TI=(NIDDM OR MODY OR t2d OR typ* two NEAR/4 diabet* OR typ?two NEAR/4 diabet* OR typ* 2 NEAR/4 diabet* OR typ* II NEAR/4 diabet* OR typ?2 NEAR/4 diabet* OR typ?II NEAR/4 diabet* OR typ* ii NEAR/4 diabet* OR typ?ii NEAR/4 diabet* OR non insulin NEAR/4 diabet* OR noninsulin NEAR/4 diabet* OR late or adult* NEAR/4 diabet* OR matur* NEAR/4 diabet* OR slow NEAR/4 diabet* OR stabl* NEAR/4 diabet* OR ketoresist* NEAR/4 diabet* OR keto* resist* NEAR/4 diabet* OR keto* prone NEAR/4 diabet*)
#5	TS=(NIDDM OR MODY OR t2d OR typ* two NEAR/4 diabet* OR typ?two NEAR/4 diabet* OR typ* 2 NEAR/4 diabet* OR typ* II NEAR/4 diabet* OR typ?2 NEAR/4 diabet* OR typ?II NEAR/4 diabet* OR typ* ii NEAR/4 diabet* OR typ?ii NEAR/4 diabet* OR non insulin NEAR/4 diabet* OR noninsulin NEAR/4 diabet* OR late or adult* NEAR/4 diabet* OR matur* NEAR/4 diabet* OR slow NEAR/4 diabet* OR stabl* NEAR/4 diabet* OR ketoresist* NEAR/4 diabet* OR keto* resist* NEAR/4 diabet* OR keto* prone NEAR/4 diabet*)
#6	#4 OR #5
#7	#3 AND #6
#8	TI=(retinopath* OR retinitis OR NPDR OR PDR OR vitre* NEAR/2 detach* OR vitre* NEAR/2 hemorrhag* retina* NEAR/2 detach* OR diabet* NEAR/3 maculopath*)
#9	TS=(retinopath* OR retinitis OR NPDR OR PDR OR vitre* NEAR/2 detach* OR vitre* NEAR/2 hemorrhag* retina* NEAR/2 detach* OR diabet* NEAR/3 maculopath*)
#10	#8 OR #9
#11	#7 AND #10

© 2023 Cioana M et al. *JAMA Network Open*.

eTable 7. Type 2 Diabetes Criteria Used Across the Included Studies

**Author, year**	**T2DM Diagnostic Criteria**	**Testing of autoantibodies**
Aulich, 2019[2]	Negative autoantibodies and without maturity onset diabetes of the young (MODY) or secondary diabetes mellitus	Y, all negative, unspecified
Amutha, 2021[3]	Absence of ketosis, or fasting C-peptide assay ≥0.6 pmol/ml and stimulated C-peptide value >1.0 pmol/ml, or good response to oral hypoglycemic agents for more than 2 years	Y, GAD positive: 8/280 (2.9%)
Bai, 2022[4]	International Classification of Diseases, and International Classification of Diseases (ICD), Ninth Revision, codes diagnosing type 2 diabetes as “non–insulin-dependent diabetes mellitus” or “type 2 diabetes”	NR
Dart, 2014[5]	CDA criteria[31]	Y, when available
Ek, 2020[6]	ADA criteria[32]	Y, when available
Eppens, 2006 (Australia)[7]	Australasian Pediatric Endocrine Group criteria[33]: negative diabetes-associated antibodies, elevated fasting insulin or C-peptide, or presence of acanthosis nigricans	Y, all negative, unspecified
Eppens, 2006 (Western Pacific)[8]	OGTT, C-peptide or insulin levels, negative diabetes-associated autoantibodies, or clinical judgement	Y, all negative, unspecified
Farah, 2006[9]	Diagnosis from medical records	NR
Ferm, 2021[10]	Diagnosis from medical records	NR
Geloneck, 2015[11]	Diagnosis from medical records	NR
Jefferies, 2012[12]	ADA criteria[32]	Y, when available
Jensen, 2021[13]	ADA criteria,[32] T2DM diagnosed in patients with negative pancreatic antibodies and fasting C-peptide ≥3.7 ng/ml or using clinical definitions	Y, GAD, IA-2, insulin: results NR
Khalil, 2019[14]	ADA criteria[32]	NR
Koziol, 2020[15]	ICD10 codes for T2DM (E11)	NR
Lee, 2007[16]	Diagnosis from medical records	NR
Newton, 2015[17]	Diagnosis from medical records	NR
Osman, 2013[18]	Clinical diagnosis based on T2DM symptoms at onset, presence of obesity, acanthosis nigricans, other features of metabolic syndrome, family history of T2DM and availability of abnormal insulin, or C-peptide levels as well as treatment given	NR
Preechasuk, 2022[19]	ADA criteria[32]	Y, when available
Porter, 2020[20]	ICD10 codes for T2D (E11)	NR
Ruhayel, 2010[21]	ISPAD criteria[34], clinical details used such as strong family history of T2DM, being of high-risk ethnicity and presence of acanthosis nigricans to support diagnosis	Y, GAD, insulin and ICA: all negative

© 2023 Cioana M et al. *JAMA Network Open*.

Schmidt, 2012[22]	Diagnosis from medical records	NR
Scott, 2006[23]	Diagnosis from medical records	NR
Shield, 2009[24]	ADA criteria,[32] and raised fasting insulin (>132 pmol/l) or fasting C-peptide concentrations (>0.6 nmol/l) and/or negative ICA, GAD, insulin antibodies, with no insulin requirement 1 year after diagnosis or a case not meeting the above criteria but in which there had been no insulin requirement for the year after diagnosis	Y, ICA, GAD, insulin: all negative (only done in unclear cases)
TODAY, 2013[25]	ADA criteria[32]	Y, GAD and tyrosine phosphatase: all negative
TODAY2, 2022[26]	ADA criteria[32]	Y, GAD and tyrosine phosphatase: all negative
Unnikrishnan, 2008[27]	No insulin requirement, no history of ketoacidosis, condition controlled on oral drugs for >1 year after	Y, GAD, all negative
Wang, 2017[28]	At least 2 T2DM diagnoses (ICD-9-CM codes 250.xx or 362.01–362.07) on separate dates	NR
Yeh, 2017[29]	Diagnosis from medical records	NR
Zuckerman Levin, 2022[30]	ADA criteria[32]	Y, Unspecified: all negative

Legend: Y: yes, NR: not reported, GAD: glutamic acid decarboxylase, CDA: Canadian Diabetes Association, ADA: American Diabetes Association, OGTT: oral glucose tolerance test, T2DM: type 2 diabetes mellitus, IA-2: islet tyrosine phosphatase 2 antibody, ICA: islet cell antibodies, ISPAD: International Society for Pediatric and Adolescent Diabetes

© 2023 Cioana M et al. *JAMA Network Open*.

eTable 8. Characteristics of Included Studies

Author, Year (Country)	Duration of diabetes (years)	Diabetic retinopathy prevalence No. (%)	Sample size	Sex distribution No. (%)	Racial distribution No. (%)	Subgroup by sex or ethnic group No. (%)	Prevalence of obesity No. (%)	Hypertension prevalence No. (%)	Mean HbA1c (%)
**Eppens et al,[42] 2006 (Western Pacific)**	2.3 (1.4-3.6)[b,d]	2 (0.6)	284	NR	NR	NR	106 (32.0)[d]	79 (24.0)[d]	7.0 (5.9–9.9)[c,d]
**Farah et al,[43] 2006 (USA)**	1.8 (<2-15)[d]	1 (2.5)	40	NR	NR	NR	29 (72.5)	NR	9.1 ± NR
**Unnikrishnan et al,[44] 2008 (India)**	NR	0 (0.0)	36	M: 21 (58.3), F: 15 (41.7)	Indian: 36 (100.0)[e]	Indian: 0 (0.0)[e]	NR	1 (3)	8.7 ± 2.1
**Aulich et al,[45] 2019 (Australia)**	1.8 (0.3–3.3)[b,d]	2 (6.7)	30	NR	NR	NR	24 (75.0)[d]	6 (19.3)[d]	6.6 (5.6-9.2)[b,d]
**Khalil et al,[46] 2019 (Egypt)**	2.5 ± 2.0	0 (0.0)	13	M: 6 (46.2), F: 7 (53.8)	Egyptian: 13 (100.0)[e]	Egyptian: 0 (0.0)[e]	NR	NR	NR
**Ferm et al,[47] 2021 (USA)**	NR	13 (3.1)	416	NR	NR	NR		NR	NR
**Scott et al,[48] 2006 (New Zealand)**	3.0 ± 0.3	8 (7.6)	105	NR	Maori/Pacific Islander/Other: 66 (62.9), European: 39 (37.1)	NR	105 (100)	21 (20.0)	8.5 ± 0.2
**Lee et al,[49] 2007 (Japan)**	NR	10 (27.8)	36	M: 11 (31.6), F: 25 (69.4)	Japanese: 36 (100.0)[e]	M: 0 (0.0), F: 10 (40.0), Japanese: 10 (27.8)[e]	NR	NR	NR
**Osman et al,[50] 2013 (Sudan)**	NR	0 (0.0)	38	M: 17 (44.7), F: 21 (55.3)	Arab: 32 (84.2), Mixed: 4 (10.5), Non-Arab: 2 (5.3)	NR	29 (76.3)	22 (57.9)	9.1 ± NR
**Dart et al,[51] 2014 (Canada)**	4.4 (0–27.4)[c]	40 (11.7)	342	M: 129 (37.8), F: 213 (62.2)	NR	NR	NR	34 (9.9)[l]<br><br>157 (45.8)[m]	8.9 ± 3.0[d]
**Geloneck et al,[52] 2015 (USA)**	2.8 ± 2.3	0 (0.0)	32	M: 12 (37.5), F: 20 (62.5)	African American: 27 (84.4), Caucasian: 4 (12.5), Asian: 1 (3.1)	NR	NR	NR	8.9 ± 2.9

© 2023 Cioana M et al. *JAMA Network Open*.

**Newton et al,[53] 2015 (New Zealand)[a]**	0	1 (4.3)	23	NR	Samoan: 6 (26.2), Maori: 4 (17.4), Tokelauan: 4 (17.4), Cook Island Maori: 2 (8.7), Tongan: 2 (8.7), Chinese: 2 (8.7), Fijian: 1 (4.3), Indian: 1 (4.3), New Zealand European: 1 (4.3)	NR	22 (95.7)	NR	NR
**Wang et al,[54] 2017 (USA)**	3.1 (1.9-4.9)[b]	127 (7.2)	1768	M: 300 (17.0), F: 1468 (83.0)	Caucasian: 1278 (72.3), African American: 206 (11.7), Latino: 214 (12.1), Asian: 70 (4.0)	NR	NR	NR	5.6 (5.4-6.4)[d]
**Yeh et al,[55] 2017 (USA)[a]**	NR	1 (7.1)	14	NR	NR	NR	NR	11 (16.9)[l] 26 (40.0)[m]	8.5 ± NR
**Koziol et al,[56] 2020 (Poland)**	NR	79 (1.8)	4291[#]	NR	Polish: 4291 (100)[e]	Polish: 79 (1.84)[e]	NR	NR	NR
**Ek et al,[57] 2020 (Sweden)**	6.7 ± 2.8	32 (31.1)	103	M: 62 (47.7) F: 68 (52.3)	Swedish: 103 (100)[e]	Swedish: 32 (31.1)[e]	60 (46.2)	12 (13.2)[d]	7.5± NR[d]
**Porter et al,[58] 2020 (USA)**	1.1 ± 1.26	3 (6.0)	50	M: 17 (34.0) F: 33 (66.0)	Non-Hispanic White: 12 (24.0), Non-Hispanic Black: 28 (56.0), Hispanic: 6 (12.0), Unknown 4 (8.0)	NR	NR	NR	8.1 ± 2.8
**Amutha et al,[59] 2021 (India)**	5.7 (NR-NR)[b,d]	118 (27.5)	429	NR	South Indian: 429 (100)[e]	South Indian: 118 (27.5)[e]	689 (77.5) [d,f]	157 (17.7)[d]	9.8 ± 2.4[d]
**Bai et al,[60] 2022 (USA)**	0-15	17 (26.6)	64	M: 18 (28.1), F: 46 (71.9)	American Indian: 1 (1.6), Asian: 9 (14.1), Black: 12 (18.7), Hispanic: 4 (6.3), White: 35 (54.7), Mixed: 1 (1.6), Other: 1 (1.6), Unknown: 1 (1.6)	NR	NR	38 (8.2)	10.6 (2.5)

© 2023 Cioana M et al. *JAMA Network Open*.

**Eppens et al,[12] 2006 (Australia)**	1.3 (0.6-3.1)[b,d]	1 (4.0)	25	NR	NR	NR	36 (56.3)[d]	21 (36.2)[d]	7.3 (6.0–8.3)
**Shield et al,[61] 2009 (UK and Republic of Ireland)**	1[d]	0 (0.0)	55	NR	NR	NR	61 (80.3)[d]	19 (32.2)[d]	7.5 ± NR
**Ruhayel et al,[62] 2010 (Australia)**	5.17 ± 1.96[d]	4 (25.0)	16	NR	NR	NR	23 (69.7)[d]	9 (30.0)[d]	7.0 (5.3–13.6)[c]
**Jefferies et al,[63] 2012 (New Zealand)**	NR	0 (0.0)	52	M: 17 (32.7), F: 35 (67.3)	Pacific Island or Maori: 47 (90.4), Other: 5 (9.6)	NR	NR	27 (52)	9.5 ± 2.5
**Schmidt et al,[64] 2012 (Germany and Austria)**	NR	12 (1.8)	684	M: 261 (38.2), F: 423 (61.8)	German/Austrian: 482 (70.5), Other: 202 (29.5)	NR	NR	202 (29.5)	7.19 ± 2.10
**Jensen et al,[66] 2021 (USA)[a]**	7.5 ± 2.1	140 (31.3)	447	M: 77 (33.6), F: 152 (66.4)	Asian/Pacific Islander: 3 (1.3), Black: 100 (43.7), Hispanic: 51 (22.3), Native American: 19 (8.3), White: 55 (24.0), Other: 1 (0.4)	NR	NR	NR	NR
	12.4 ± 2.1	126 (55.0)	229			M: 45 (58.4), F: 81 (53.3), Asian/Pacific Islander: 1 (33.3), Black: 64 (64.0), Hispanic: 27 (52.9), Native American: 9 (47.4), White: 25 (45.5), Other: 0 (0.0)			
**Preechasuk et al,[67] 2022 (Thailand)**	5.2 (1.6-9.4)[b]	8 (9.0)	89	M: 41 (43.2), F: 54 (56.8)	Thai: 89 (100.0)[e]	Thai: 8 (9.0)[e]	79 (83.2)	35 (36.8)	NR
**TODAY Study Group et al,[68] 2013 (USA)**	4.9 ± 1.5	71 (13.7)	517	M: 184 (35.6), F: 333 (64.4)	NR	NR	517 (100.0)[f]	NR	7.1 ± 1.7
**TODAY2 et al,[65] 2021 (USA)**	12.0 ± 1.5	210 (50.0)	420	NR	NR	NR	NR	224 (61.0)	7.9 ± 1.9
**Zuckerman Levin et al,[69]**	At presentation: 0	At presentation: 4 (1.9)	At presentation: 216	M: 151 (39.8), F: 228 (60.2)	Israeli Jews: 221 (58.3), Israeli	NR	NR	128 (33.7)	8.8 (2.5)

© 2023 Cioana M et al. *JAMA Network Open*.

**2022 (Israel)**	At follow-up: 2.9 ± 2.1	At follow-up: 5 (4.6)	At follow-up: 108		Arabs: 158 (41.7)

Footnote: USA: United States of America, UK: United Kingdom, NR: not reported, NPDR: non-proliferative diabetic retinopathy, PDR: proliferative diabetic retinopathy, M: males, F: females; a: abstract only; age and duration reported as range, mean, mean ± standard deviation, mean (range), subgroup: n(%), b: median (interquartile range), c: median (range); d: represents value for whole cohort in study, not just patients screened for retinopathy, e: race assumed to match country of study, f: includes patients with obesity and overweight, TODAY, Treatment Options for Type 2 Diabetes in Adolescents and Youth Study.

The references in the table correspond to the reference sequence in the main paper, and not the references in this supplement.

[#]This number was reported as the number of observations, and it was not clear if these were unique patients. The calculation of the prevalence with the inclusion and exclusion of the study did not substantially alter the results.

© 2023 Cioana M et al. *JAMA Network Open*.

eTable 9. Comparison of Meta-analysis Results Using Freeman-Tukey Double Arcsine Transformation and Generalized Linear Mixed-Effects Model With Confidence and Prediction Intervals

Parameter	Freeman-Tukey double arcsine transformation			Generalized linear mixed-effects model		
	**Value**	**95%CI**	**95%PI**	**Value**	**95%CI**	**95%PI**
DR prevalence	6.99%	3.75-11.00	0.00-33.99	5.25%	2.92-9.28	0.30-50.75
Study Design						
Cross-sectional	1.14%	0.05-3.07	0.00-7.10	1.93%	0.89-4.15	0.33-10.35
Retrospective cohort	11.29%	5.82-18.10	0.00-41.76	10.20%	5.44-18.30	1.04-55.22
Prospective cohort	6.52%	0.95-15.66	0.00-50.92	4.65%	1.58-12.90	0.11-68.20
DR severity						
Minimal-moderate NPDR	11.16%	1.52-27.21	0.00-79.59	8.54%	3.03-21.84	0.20-81.17
Severe NPDR	2.57%	0.58-5.69	N/A	2.36%	1.34-4.10	N/A
PDR	2.43%	0.04-7.47	0.00-42.93	1.52%	0.26-8.41	0.00-98.61
Macular edema	3.09%	1.64-4.91	N/A	3.31%	2.03-5.33	N/A
Method						
Fundoscopy	0.47%	0.00-3.30	0.00-5.88	1.48%	0.37-5.73	0.16-12.67
Seven-field stereoscopic fundus photography	13.55%	5.43-24.29	0.00-70.20	13.16%	6.16-25.90	0.52-81.26
Diabetes duration						
0-2.5 years	1.78%	0.25-4.20	0.00-8.90	2.34%	1.22-4.44	0.81-6.56
2.5-5 years	5.08%	1.04-11.22	0.00-32.90	5.96%	2.47-13.67	0.50-44.57
>5 years	28.83%	15.97-43.63	0.00-83.46	27.78%	16.29-43.20	2.96-82.90
Race						
White/Middle Eastern	24.07%	6.26-47.91	0.00-100.0	22.85%	5.41-60.52	0.00-100.0
Asian	13.31%	2.49-30.05	0.00-95.14	11.84%	3.42-33.72	0.04-97.88

CI: confidence interval, PI: prediction interval.

© 2023 Cioana M et al. *JAMA Network Open*.

eTable 10. Risk of Bias of Included Studies

Author, year	External Validity Items				Internal Validity Items						Overall Score	Overall Risk of Bias	OCEBM Level of Evidence
	1	2	3	4	5	6	7	8	9	10			
Aulich, 2019[2]	0	1	0	1	1	1	1	1	1	1	8	moderate	3
Amutha, 2021[3]	0	1	1	0	1	1	1	1	1	1	8	moderate	1
Bai, 2022[4]	0	1	1	1	1	1	1	1	1	1	9	low	1
Dart, 2014[5]	0	1	1	1	1	1	1	1	1	1	8	moderate	1
Ek, 2020[6]	1	1	1	1	1	1	1	1	1	1	10	low	1
Eppens, 2006 (Australia)[7]	0	1	1	0	1	1	1	1	1	1	8	moderate	1
Eppens, 2006 (Western Pacific)[8]	1	0	1	1	1	0	0	1	1	1	7	moderate	2
Farah, 2006[9]	0	0	0	1	1	1	1	1	1	0	6	moderate	3
Ferm, 2021[10]	1	1	1	1	1	1	1	1	1	1	10	low	1
Geloneck, 2015[11]	0	1	1	1	1	1	1	1	1	1	9	low	2
Jefferies, 2012[12]	0	1	1	1	1	0	0	1	1	1	7	moderate	1
Jensen, 2021[13]	1	1	0	0	1	1	1	1	1	1	8	moderate	3
Khalil, 2019[14]	0	1	1	0	1	1	1	1	1	1	8	moderate	2
Koziol, 2020[15]	1	1	1	1	1	1	1	0	1	1	9	low	1
Lee, 2007[16]	0	0	0	1	1	0	0	1	1	1	5	high	3
Newton, 2015[17]	0	1	1	0	1	1	1	1	1	1	8	moderate	2
Osman, 2013[18]	0	1	1	1	1	1	1	1	1	1	8	moderate	2
Preechasuk, 2022[19]	0	1	1	1	1	0	0	1	1	1	7	moderate	1
Porter, 2020[20]	0	1	1	1	1	1	1	1	1	1	9	low	1
Ruhayel, 2010[21]	0	1	1	0	1	1	1	1	1	1	7	moderate	2
Schmidt, 2012[22]	1	1	1	0	1	0	0	0	1	1	6	moderate	1
Scott, 2006[23]	0	1	1	1	1	1	1	0	1	1	8	moderate	2
Shield, 2009[24]	1	1	1	1	1	0	0	0	1	1	7	moderate	1
TODAY, 2013[25]	1	1	0	0	1	1	1	1	1	1	8	moderate	3
TODAY2, 2022[26]	1	1	0	0	1	1	1	1	1	1	8	moderate	3
Unnikrishnan, 2008[27]	1	1	1	1	1	1	1	0	1	1	9	low	2
Wang, 2017[28]	1	1	1	1	1	1	1	0	1	1	9	low	1
Yeh, 2017[29]	0	1	1	0	1	1	1	1	1	1	8	moderate	2
Zuckerman Levin, 2022[30]	1	1	1	0	1	0	0	1	1	1	7	moderate	1

Legend: 0: no, 1: yes, overall risk of bias: low (score >8), moderate (score 6-8), or high (score ≤5). Items scored: 1) Was the study's target population a close representation of the national population in relation to relevant variables, e.g., age, sex?; 2) Was the sampling frame a true or close representation of the target population?; 3) Was some form of random selection used to select the sample, OR, was a census undertaken?; 4) Was the likelihood of non-response bias minimal?; 5) Were data collected directly from the subjects (as opposed to a proxy)?; 6) Was an acceptable case definition used in the study?; 7) Had the study instrument that measured the parameter of interest (e.g., prevalence of comorbidity) been tested for reliability and validity (if necessary)?; 8) Was the same mode of data collection used for all subjects?; 9) Was the length of the shortest prevalence period for the parameter of interest appropriate?; 10) Were the numerator(s) and denominator(s) for the parameter of interest appropriate?

© 2023 Cioana M et al. *JAMA Network Open*.

eTable 11. Results of Sensitivity Analysis

Studies removed	Pooled prevalence estimate (%, 95% CI)	Heterogeneity (I^2, χ^2 P)
**Conference abstracts**[13,17,29]	5.94% (95% CI 3.24-9.28)	96%, <0.001
**Different definition**[4,5,8,12,15–24,28,30]	8.88% (95% CI 3.18-16.71)	96%, <0.001
**Sample size <50**[2,7,9,11,14,16–18,21,27,29,35]	7.54% (95% CI 3.65-12.50)	98%, <0.001
**Patients over 18 years old**[3,4,6,12–16,18–20,23,25,27,28]	3.06% (95% CI 1.04-5.84)	83%, <0.001
**High risk of bias**[16]	6.18% (95% CI 3.21-9.90)	97%, <0.001

© 2023 Cioana M et al. *JAMA Network Open*.

eFigure 1. PRISMA Flow Diagram Illustrating Study Selection

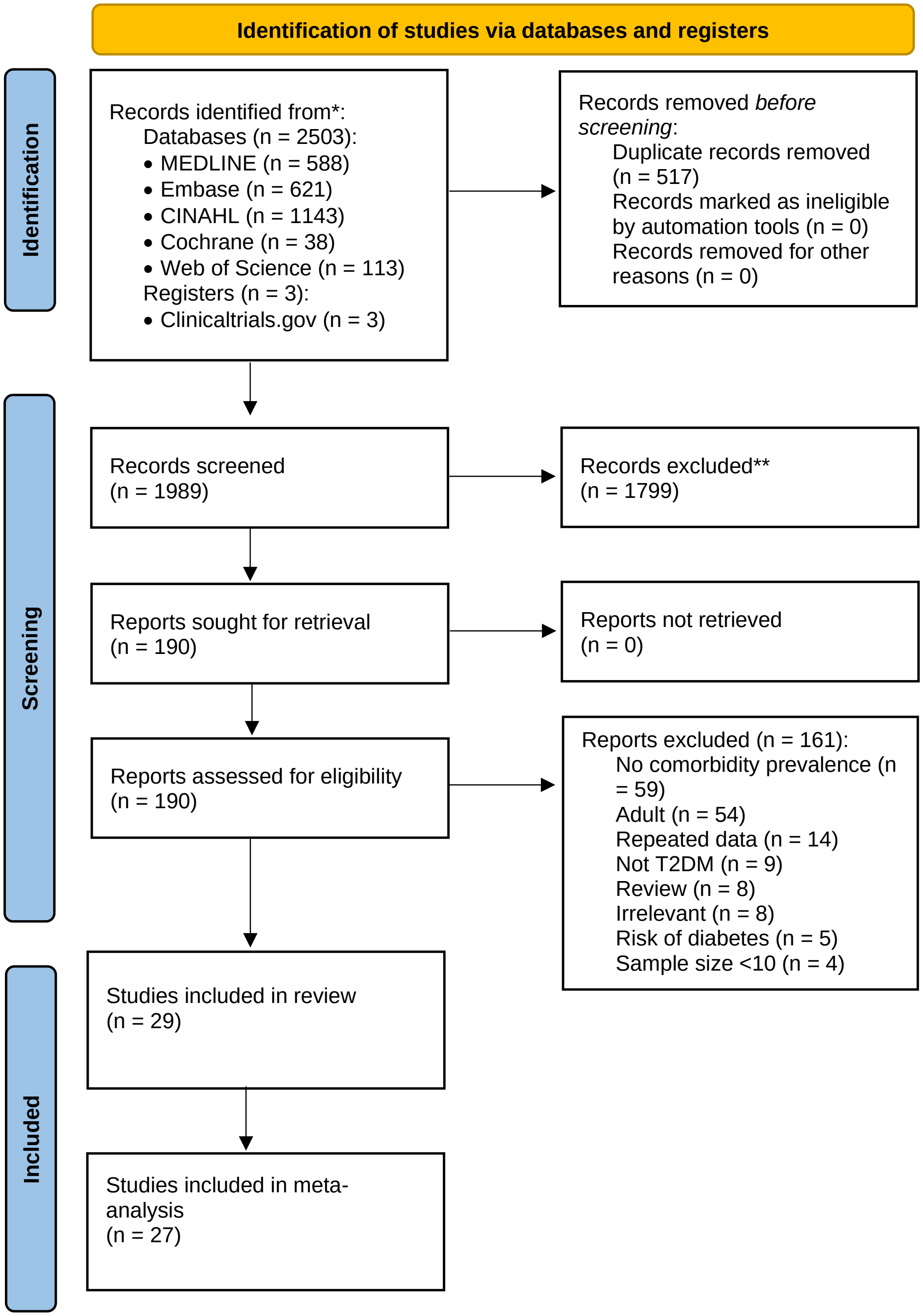

© 2023 Cioana M et al. *JAMA Network Open*.

eFigure 2. Forest Plot of Prevalence of Diabetic Retinopathy in Pediatric Type 2 Diabetes by Diabetes Duration in Prospective Cohort Studies

Study	Cases	Total	Weight	Prevalence (%) [95% CI]	Events per 100 observations IV, Random, 95% CI
**Diabetes duration = 0.0−2.5 years**					
Shield, 2009 (UK and Republic of Ireland)	0	55	12.2%	0.00 [0.00; 3.10]	
Zuckerman Levin, 2022 (Israel)	4	216	12.9%	1.85 [0.40; 4.17]	
**Total (95% CI)**		**271**	**25.1%**	**1.11 [0.04; 3.06]**	
Heterogeneity: $Tau^2 = 0.0002$; $Chi^2 = 1.06$, df = 1 (P = 0.30); $I^2 = 5\%$					
**Diabetes duration = 2.5−5.0 years**					
TODAY Study Group, 2013 (USA)	71	517	13.0%	13.73 [10.89; 16.84]	
Zuckerman Levin, 2022 (Israel)	5	108	12.7%	4.63 [1.32; 9.55]	
**Total (95% CI)**		**625**	**25.7%**	**9.04 [2.24; 19.55]**	
Heterogeneity: $Tau^2 = 0.0105$; $Chi^2 = 8.54$, df = 1 (P < 0.01); $I^2 = 88\%$					
**Diabetes duration = >5.0 years**					
Ruhayel, 2010 (Australia)	4	16	10.5%	25.00 [6.31; 49.54]	
Jensen, 2021 (USA)	140	447	13.0%	31.32 [27.10; 35.70]	
Preechasuk, 2022 (Thailand)	8	89	12.6%	8.99 [3.79; 15.94]	
TODAY2 Study Group, 2022 (USA)	210	420	13.0%	50.00 [45.22; 54.78]	
**Total (95% CI)**		**972**	**49.2%**	**28.14 [12.84; 46.45]**	
Heterogeneity: $Tau^2 = 0.0324$; $Chi^2 = 78.01$, df = 3 (P < 0.01); $I^2 = 96\%$					
**Total (95% CI)**		**1868**	**100.0%**	**13.32 [3.51; 27.66]**	

Heterogeneity: $Tau^2 = 0.0616$; $Chi^2 = 374.27$, df = 7 (P < 0.01); $I^2 = 98\%$

Test for subgroup differences: $Chi^2 = 21.62$, df = 2 (P < 0.01)

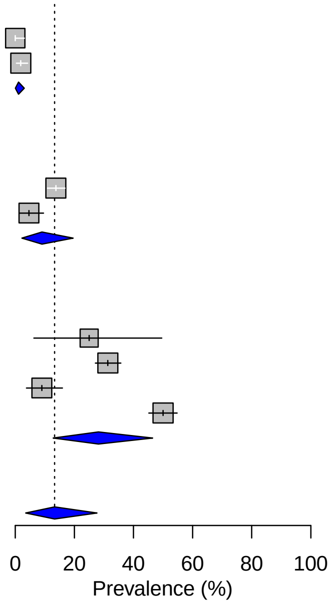

© 2023 Cioana M et al. *JAMA Network Open*.

eFigure 3. Forest Plot of Prevalence of Diabetic Retinopathy in Pediatric Type 2 Diabetes by Diabetes Duration in Retrospective Cohort Studies

Study	Cases	Total	Weight	Prevalence (%) [95% CI]
**Diabetes duration = 0.0-2.5 years**				
Newton, 2015 (New Zealand)	1	23	14.1%	4.35 [0.00; 17.73]
Porter, 2020 (USA)	3	50	25.9%	6.00 [0.78; 14.65]
**Total (95% CI)**		**73**	**40.0%**	**5.30 [0.87; 12.19]**
Heterogeneity: $Tau^2 = 0$; $Chi^2 = 0.01$, df = 1 (P = 0.90); $I^2 = 0\%$				
**Diabetes duration = 2.5-5.0 years**				
Scott, 2006 (New Zealand)	8	105	41.6%	7.62 [3.20; 13.58]
Geloneck, 2015 (USA)	0	32	18.4%	0.00 [0.00; 5.31]
**Total (95% CI)**		**137**	**60.0%**	**3.09 [0.00; 14.06]**
Heterogeneity: $Tau^2 = 0.0149$; $Chi^2 = 3.96$, df = 1 (P = 0.05); $I^2 = 75\%$				
**Total (95% CI)**		**210**	**100.0%**	**4.52 [1.36; 8.97]**
Heterogeneity: $Tau^2 = 0.0019$; $Chi^2 = 4.04$, df = 3 (P = 0.26); $I^2 = 26\%$				
Test for subgroup differences: $Chi^2 = 0.26$, df = 1 (P = 0.61)				

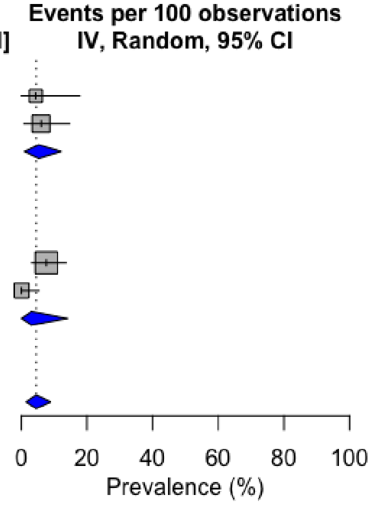

© 2023 Cioana M et al. *JAMA Network Open*.

eFigure 4. Forest Plot of Prevalence of Diabetic Retinopathy in Pediatric Type 2 Diabetes by Diabetes Duration in All Studies

Study	Cases	Total	Weight	Prevalence (%) [95% CI]
**Diabetes duration = 0.0−2.5 years**				
Farah, 2006 (USA)	1	40	6.5%	2.50 [0.00; 10.42]
Shield, 2009 (UK and Republic of Ireland)	0	55	6.7%	0.00 [0.00; 3.10]
Newton, 2015 (New Zealand)	1	23	6.1%	4.35 [0.00; 17.73]
Porter, 2020 (USA)	3	50	6.7%	6.00 [0.78; 14.65]
Zuckerman Levin, 2022 (Israel)	4	216	7.1%	1.85 [0.40; 4.17]
**Total (95% CI)**		**384**	**33.1%**	**1.78 [0.25; 4.20]**
Heterogeneity: $Tau^2 = 0.0013$; $Chi^2 = 5.23$, df = 4 (P = 0.26); $I^2 = 23\%$				
**Diabetes duration = 2.5−5.0 years**				
Scott, 2006 (New Zealand)	8	105	7.0%	7.62 [3.20; 13.58]
TODAY Study Group, 2013 (USA)	71	517	7.2%	13.73 [10.89; 16.84]
Geloneck, 2015 (USA)	0	32	6.4%	0.00 [0.00; 5.31]
Khalil, 2019 (Egypt)	0	13	5.5%	0.00 [0.00; 12.82]
Zuckerman Levin, 2022 (Israel)	5	108	7.0%	4.63 [1.32; 9.55]
**Total (95% CI)**		**775**	**32.9%**	**5.08 [1.04; 11.22]**
Heterogeneity: $Tau^2 = 0.0102$; $Chi^2 = 20.45$, df = 4 (P < 0.01); $I^2 = 80\%$				
**Diabetes duration = >5.0 years**				
Ruhayel, 2010 (Australia)	4	16	5.7%	25.00 [6.31; 49.54]
Ek, 2020 (Sweden)	32	103	7.0%	31.07 [22.46; 40.38]
Jensen, 2021 (USA)	140	447	7.2%	31.32 [27.10; 35.70]
Preechasuk, 2022 (Thailand)	8	89	6.9%	8.99 [3.79; 15.94]
TODAY Study Group, 2022 (USA)	210	420	7.2%	50.00 [45.22; 54.78]
**Total (95% CI)**		**1075**	**33.9%**	**28.83 [15.97; 43.63]**
Heterogeneity: $Tau^2 = 0.0265$; $Chi^2 = 79.22$, df = 4 (P < 0.01); $I^2 = 95\%$				
**Total (95% CI)**		**2234**	**100.0%**	**9.58 [3.16; 18.56]**
Heterogeneity: $Tau^2 = 0.0561$; $Chi^2 = 442.85$, df = 14 (P < 0.01); $I^2 = 97\%$				
Test for subgroup differences: $Chi^2 = 23.49$, df = 2 (P < 0.01)				

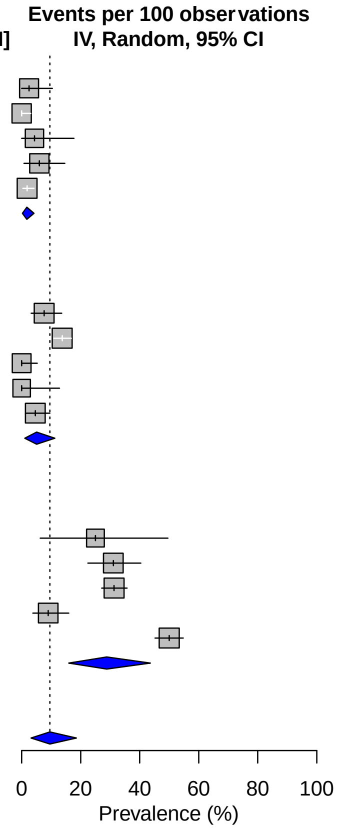

© 2023 Cioana M et al. *JAMA Network Open*.

eFigure 5. Forest Plot of the Odds Ratio of Diabetic Retinopathy in Pediatric Type 2 Diabetes by Sex

	Males		Females			Odds Ratio	
**Study or Subgroup**	**Events**	**Total**	**Events**	**Total**	**Weight**	**M-H, Random, 95% CI**	**Year**
Lee 2007	0	11	10	25	38.3%	0.06 [0.00, 1.21]	2007
Jensen 2021	45	77	81	152	61.7%	1.23 [0.71, 2.15]	2021
**Total (95% CI)**		**88**		**177**	**100.0%**	**0.40 [0.02, 7.21]**	
Total events	45		91				

Heterogeneity: $Tau^2 = 3.46$; $Chi^2 = 3.98$, df = 1 (P = 0.05); $I^2 = 75\%$
Test for overall effect: Z = 0.62 (P = 0.53)

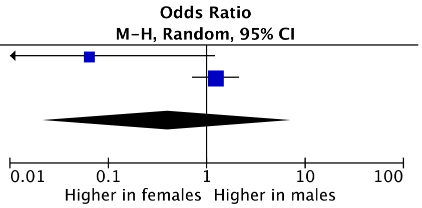

© 2023 Cioana M et al. *JAMA Network Open*.

eFigure 6. Forest Plot Illustrating Prevalence of Diabetic Retinopathy in Pediatric Type 2 Diabetes by Race

Study	Cases	Total	Weight	Prevalence (%) [95% CI]
**Racial Group = White/Middle Eastern**				
Khalil, 2019 (Egypt)	0	13	10.5%	0.00 [0.00; 12.82]
Ek, 2020 (Sweden)	32	103	15.6%	31.07 [22.46; 40.38]
Jensen, 2021 (USA)	25	55	14.6%	45.45 [32.43; 58.80]
**Total (95% CI)**		**171**	**40.7%**	**24.07 [6.26; 47.91]**
Heterogeneity: $Tau^2 = 0.0384$; $Chi^2 = 16.06$, df = 2 (P < 0.01); $I^2 = 88\%$				
**Racial Group = Asian**				
Lee, 2007 (Japan)	10	36	13.7%	27.78 [14.18; 43.70]
Unnikrishnan, 2008 (India)	0	36	13.7%	0.00 [0.00; 4.72]
Amutha, 2021 (India)	118	429	16.5%	27.51 [23.38; 31.84]
Preechasuk, 2022 (Thailand)	8	89	15.4%	8.99 [3.79; 15.94]
**Total (95% CI)**		**590**	**59.3%**	**13.31 [2.49; 30.05]**
Heterogeneity: $Tau^2 = 0.0381$; $Chi^2 = 43.03$, df = 3 (P < 0.01); $I^2 = 93\%$				
**Total (95% CI)**		**761**	**100.0%**	**17.56 [7.84; 29.85]**
Heterogeneity: $Tau^2 = 0.0308$; $Chi^2 = 66.40$, df = 6 (P < 0.01); $I^2 = 91\%$				
Test for subgroup differences: $Chi^2 = 0.76$, df = 1 (P = 0.38)				

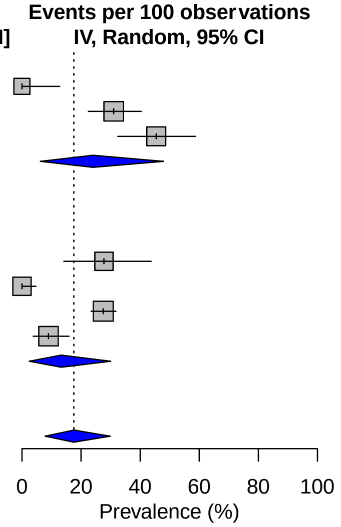

© 2023 Cioana M et al. *JAMA Network Open*.

eFigure 7. Forest Plot Showing Mean Difference in HbA_{1c} in Participants With vs Without Diabetic Retinopathy

Study or Subgroup	DR Mean	DR SD	DR Total	No DR Mean	No DR SD	No DR Total	Weight	Mean Difference IV, Random, 95% CI
Ferm, 2021 (USA)	10.3	2.4	13	9.2	2.1	403	9.9%	1.10 [−0.22, 2.42]
TODAY, 2013 (USA)	8.3	1.77	71	6.9	1.6	446	90.1%	1.40 [0.96, 1.84]
**Total (95% CI)**			**84**			**849**	**100.0%**	**1.37 [0.95, 1.79]**

Heterogeneity: $Tau^2 = 0.00$; $Chi^2 = 0.18$, df = 1 (P = 0.67); $I^2 = 0\%$
Test for overall effect: Z = 6.46 (P < 0.00001)

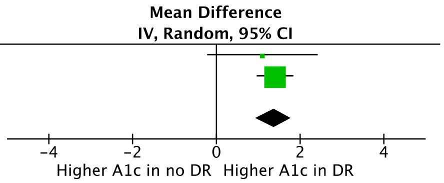

© 2023 Cioana M et al. *JAMA Network Open*.

eFigure 8. Funnel Plot Examining Publication Bias for Diabetic Retinopathy Prevalence Outcome

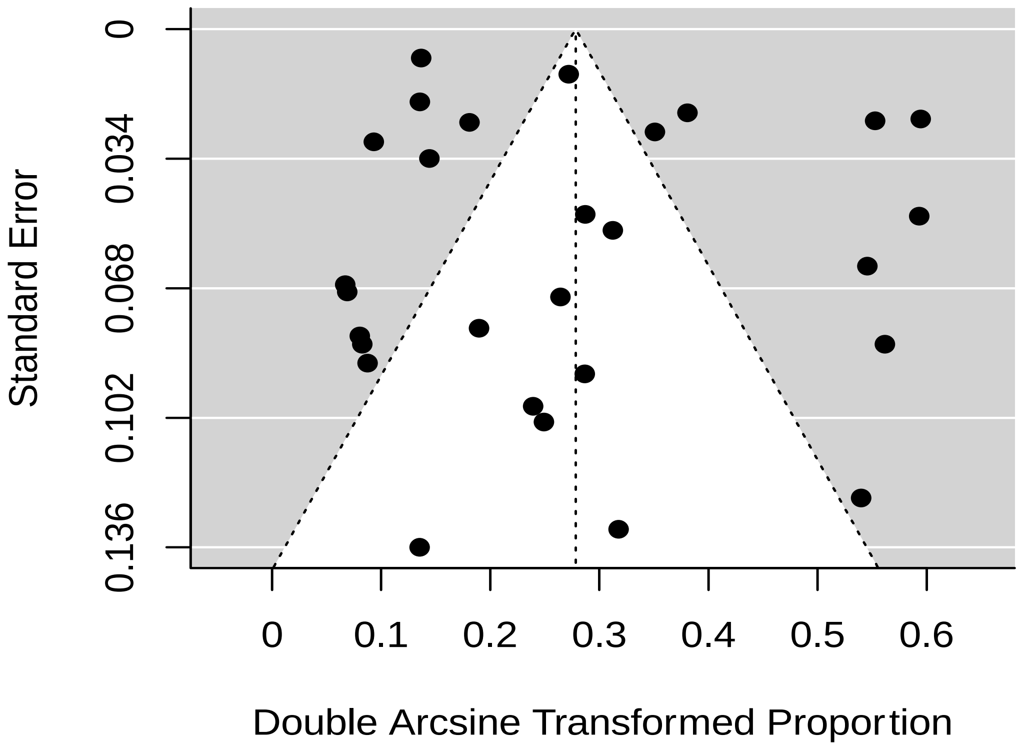

© 2023 Cioana M et al. *JAMA Network Open*.

eReferences

1. Stroup DF, Berlin JA, Morton SC, et al. Meta-analysis of Observational Studies in Epidemiology: A Proposal for Reporting. *JAMA*. 2000;283(15):2008-2012. doi:10.1001/jama.283.15.2008

2. Aulich J, Cho YH, Januszewski AS, et al. Associations between circulating inflammatory markers, diabetes type and complications in youth. *Pediatr Diabetes*. 2019;20(8):1118-1127. doi:10.1111/pedi.12913

3. Amutha A, Ranjit U, Anjana RM, et al. Clinical profile and incidence of microvascular complications of childhood and adolescent onset type 1 and type 2 diabetes seen at a tertiary diabetes center in India. *Pediatric Diabetes*. 2021;22(1):67-74. doi:https://doi.org/10.1111/pedi.13033

4. Bai P, Barkmeier AJ, Hodge DO, Mohney BG. Ocular Sequelae in a Population-Based Cohort of Youth Diagnosed With Diabetes During a 50-Year Period. *JAMA Ophthalmology*. Published online December 2, 2021. doi:10.1001/jamaophthalmol.2021.5052

5. Dart AB, Martens PJ, Rigatto C, Brownell MD, Dean HJ, Sellers EA. Earlier Onset of Complications in Youth With Type 2 Diabetes. *Diabetes Care*. 2014;37(2):436-443. doi:10.2337/dc13-0954

6. Ek AE, Samuelsson U, Janson A, Carlsson A, Elimam A, Marcus C. Microalbuminuria and retinopathy in adolescents and young adults with type 1 and type 2 diabetes. *Pediatric Diabetes*. 2020;21(7):1310-1321. doi:https://doi.org/10.1111/pedi.13074

7. Eppens MC, Craig ME, Cusumano J, et al. Prevalence of diabetes complications in adolescents with type 2 compared with type 1 diabetes. *Diabetes Care*. 2006;29(6):1300-1306. doi:10.2337/dc05-2470

8. Eppens MC, Craig ME, Jones TW, et al. Type 2 diabetes in youth from the Western Pacific region: glycaemic control, diabetes care and complications. *Current Medical Research and Opinion*. 2006;22(5):1013-1020. doi:10.1185/030079906X104795

9. Farah SE, Wals KT, Friedman IB, Pisacano MA, DiMartino-Nardi J. Prevalence of Retinopathy and Microalbuminuria in Pediatric Type 2 Diabetes Mellitus. *Journal of Pediatric Endocrinology and Metabolism*. 2006;19(7). doi:10.1515/JPEM.2006.19.7.937

10. Ferm ML, DeSalvo DJ, Prichett LM, Sickler JK, Wolf RM, Channa R. Clinical and Demographic Factors Associated With Diabetic Retinopathy Among Young Patients With Diabetes. *JAMA Netw Open*. 2021;4(9):e2126126. doi:10.1001/jamanetworkopen.2021.26126

11. Geloneck MM, Forbes BJ, Shaffer J, Ying G shuang, Binenbaum G. Ocular complications in children with diabetes mellitus. *Ophthalmology*. 2015;122(12):2457-2464. doi:10.1016/j.ophtha.2015.07.010

12. Jefferies C, Carter P, Reed PW, et al. The incidence, clinical features, and treatment of type 2 diabetes in children <15 yr in a population-based cohort from Auckland, New Zealand, 1995–2007. *Pediatr Diabetes*. 2012;13(4):294-300. doi:10.1111/j.1399-5448.2012.00851.x

© 2023 Cioana M et al. *JAMA Network Open*.

13. JENSEN ET, RIGDON J, REZAEI K, et al. 1032-P: Prevalence of Diabetic Retinopathy in Youth-Onset Type 1 and Type 2 Diabetes: The SEARCH for Diabetes in Youth Study. *Diabetes*. 2021;70(Supplement_1):1032-P. doi:10.2337/db21-1032-P

14. Khalil SA, Megallaa MH, Rohoma KH, et al. Prevalence of Chronic Diabetic Complications in Newly Diagnosed versus Known Type 2 Diabetic Subjects in a Sample of Alexandria Population, Egypt. *Curr Diabetes Rev*. 2019;15(1):74-83. doi:10.2174/1573399814666180125100917

15. Kozioł M, Nowak MS, Udziela M, Piątkiewicz P, Grabska-Liberek I, Szaflik JP. First nation-wide study of diabetic retinopathy in Poland in the years 2013–2017. *Acta Diabetol*. 2020;57(10):1255-1264. doi:10.1007/s00592-020-01540-6

16. Lee Z, Sato Y, Urakami T. [Relationship between retinopathy development and systemic factors in type 2 childhood diabetes]. *Nippon Ganka Gakkai Zasshi*. 2007;111(5):397-400.

17. Newton K, Stanley J, Wiltshire E. Audit of type 2 diabetes in youth in Wellington, New Zealand 2001–2013. *Pediatric Diabetes*. 2015;16(Suppl 21):50-150. doi:10.1111/pedi.12309

18. Osman HAM, Elsadek N, Abdullah MA. Type 2 diabetes in Sudanese children and adolescents. *Sudan J Paediatr*. 2013;13(2):17-23.

19. Preechasuk L, Tantasuwan S, Likitmaskul S, et al. Clinical Characteristics, Glycemic Control, and Microvascular Complications Compared Between Young-Onset Type 1 and Type 2 Diabetes Patients at Siriraj Hospital – A Tertiary Referral Center. *Diabetes Metab Syndr Obes*. 2022;15:1375-1387. doi:10.2147/DMSO.S354787

20. Porter M, Channa R, Wagner J, Prichett L, Liu TYA, Wolf RM. Prevalence of diabetic retinopathy in children and adolescents at an urban tertiary eye care center. *Pediatric Diabetes*. 2020;21(5):856-862. doi:https://doi.org/10.1111/pedi.13037

21. Ruhayel SD, James RA, Ehtisham S, Cameron FJ, Werther GA, Sabin MA. An observational study of type 2 diabetes within a large Australian tertiary hospital pediatric diabetes service. *Pediatric Diabetes*. 2010;11(8):544-551. doi:10.1111/j.1399-5448.2010.00647.x

22. Schmidt F, Kapellen TM, Wiegand S, et al. Diabetes Mellitus in Children and Adolescents with Genetic Syndromes. *Exp Clin Endocrinol Diabetes*. 2012;120(10):579-585. doi:10.1055/s-0032-1306330

23. Scott A, Toomath R, Bouchier D, et al. First national audit of the outcomes of care in young people with diabetes in New Zealand: high prevalence of nephropathy in Maori and Pacific Islanders. *N Z Med J*. 2006;119(1235):U2015.

24. Shield JPH, Lynn R, Wan KC, Haines L, Barrett TG. Management and 1 year outcome for UK children with type 2 diabetes. *Archives of Disease in Childhood*. 2009;94(3):206-209. doi:10.1136/adc.2008.143313

25. TODAY Study Group. Retinopathy in Youth With Type 2 Diabetes Participating in the TODAY Clinical Trial. *Diabetes Care*. 2013;36(6):1772-1774. doi:10.2337/dc12-2387

© 2023 Cioana M et al. *JAMA Network Open*.

26. TODAY Study Group. Development and Progression of Diabetic Retinopathy in Adolescents and Young Adults With Type 2 Diabetes: Results From the TODAY Study. *Diabetes Care*. 2022;45(5):1049-1055. doi:10.2337/dc21-1072

27. Unnikrishnan A. G., Bhatia Eesh, Bhatia Vijayalakshmi, et al. Type 1 Diabetes versus Type 2 Diabetes with Onset in Persons Younger than 20 Years of Age. *Annals of the New York Academy of Sciences*. 2008;1150(1):239-244. doi:10.1196/annals.1447.056

28. Wang SY, Andrews CA, Herman WH, Gardner TW, Stein JD. Incidence and Risk Factors for Developing Diabetic Retinopathy Among Youth with Type 1 and Type 2 Diabetes Throughout the United States. *Ophthalmology*. 2017;124(4):424-430. doi:10.1016/j.ophtha.2016.10.031

29. Yeh T, Bernardo J. Complications of type 2 diabetes in adolescent patients: The Rhode Island Hospital experience compared to the TODAY study. *Hormone Research in Paediatrics*. 2017;88(Supplement 1):626.

30. Zuckerman Levin N, Cohen M, Phillip M, et al. Youth-onset Type 2 diabetes in Israel: A national cohort. *Pediatric Diabetes*. 2022;n/a(n/a). doi:10.1111/pedi.13351

31. Canadian Diabetes Association. Clinical practice guidelines for the prevention and management of diabetes in Canada. *Canadian Journal of Diabetes*. 2008;32.

32. American Diabetes Association. Report of the Expert Committee on the diagnosis and classification of diabetes Mellitus. *Diabetes Care*. 2000;23:S4-S19.

33. Australasian Paediatric Endocrine Group, Department of Health and Ageing. Clinical Practice Guidelines: Type 1 Diabetes In Children and Adolescents: (509522012-001). Published online 2004. doi:10.1037/e509522012-001

34. Rosenbloom AL, Silverstein JH, Amemiya S, Zeitler P, Klingensmith GJ, International Society for Pediatric and Adolescent Diabetes. ISPAD Clinical Practice Consensus Guidelines 2006-2007. Type 2 diabetes mellitus in the child and adolescent. *Pediatr Diabetes*. 2008;9(5):512-526. doi:10.1111/j.1399-5448.2008.00429.x

35. Mayer-Davis EJ, Davis C, Saadine J, et al. Diabetic retinopathy in the SEARCH for Diabetes in Youth Cohort: a pilot study. *Diabet Med*. 2012;29(9):1148-1152. doi:10.1111/j.1464-5491.2012.03591.x

© 2023 Cioana M et al. *JAMA Network Open*.
