# Supplementary material for: Global Prevalence of Diabetic Retinopathy in Pediatric Type 2 Diabetes: A Systematic Review and Meta-analysis
Source: JAMA Netw Open. 2023 Mar 17;6(3):e231887. doi: 10.1001/jamanetworkopen.2023.1887 (PMC10024209; doi:10.1001/jamanetworkopen.2023.1887)
Supplement: Supplement 2. — Data Sharing Statement [file jamanetwopen-e231887-s002.pdf]

Data Sharing Statement

Cioana. Global Prevalence of Diabetic Retinopathy in Pediatric Type 2 Diabetes. *JAMA Netw Open*. Published March 17, 2023. doi:10.1001/jamanetworkopen.2023.1887

Data
Data available: Yes
Data types: Deidentified participant data
How to access data: Table 1, supplementary file
When available: With publication

Supporting Documents
Document types: None

Additional Information
Who can access the data: all data will be published with manuscript
Types of analyses: for any purpose
Mechanisms of data availability: all data will be published with manuscript
